# Adeno‐associated virus‐based rescue of *Myo7a* expression restores hair‐cell function and improves hearing thresholds in a *USH1B* mouse strain

**DOI:** 10.1113/JP289526

**Published:** 2025-09-27

**Authors:** Ana E. Amariutei, Samuel Webb, Adam J. Carlton, Andrew O'Connor, Anna Underhill, Jing‐Yi Jeng, Sarah A. Hool, Alice Zanella, Matthew Hool, Marie‐José Lecomte, Stuart L. Johnson, Saaid Safieddine, Walter Marcotti

**Affiliations:** ^1^ School of Biosciences University of Sheffield Sheffield UK; ^2^ Université Paris Cité, Institut Pasteur, AP‐HP, INSERM, CNRS, Fondation Pour l'Audition, Institut de l'Audition, IHU reConnect Paris France; ^3^ Neuroscience Institute University of Sheffield Sheffield UK

**Keywords:** cochlea, deafness, gene‐based therapy, hair cell, ion channels, mechanoelectrical transduction, myosin motor, ribbon synapses

## Abstract

**Abstract:**

Mutations in *MYO7A*, the gene encoding the unconventional myosin 7a, cause hereditary deafness in mice and humans. In the cochlea, MYO7A is present in the sensory hair cells from embryonic stages of development, and plays a critical role in the development and maintenance of the mechanosensitive hair bundles composed of actin‐rich stereocilia. *Shaker‐1* mutant mice (*Myo7a^Sh1/Sh1^*), the murine model of Usher 1B syndrome, exhibit a progressive loss of the stereocilia, subsequent degeneration of the sensory epithelium and ultimately profound deafness. In addition to the hair bundle defects, we found that the *shaker‐1* mutation prevented both inner hair cells (IHCs) and outer hair cells (OHCs) from acquiring their fully mature basolateral current profile. Delivering exogenous *Myo7a* to newborn *Myo7a^Sh1/Sh1^* mice using dual‐adeno‐associated virus 8 (AAV8)‐*Myo7a* or dual‐AAV9‐PhP.eB‐*Myo7a*, which primarily target IHCs, led to a substantial rescue of their hair bundle structure. The rescued bundles regained their ability to generate mechanoelectrical transducer (MET) currents in response to fluid jet displacement. Although the average MET current was smaller than in control IHCs, the normal resting open probability of the MET channel was fully restored. The IHCs of the treated cochlea also regained a mature basolateral membrane current profile. Functionally, rescue of the IHC structure and function, but not that of OHCs, leads to an average improvement of 20–30 dB in hearing thresholds across most frequencies. These results support dual AAV‐induced gene replacement therapy as an effective strategy to recover hair‐cell function in *Myo7a^Sh1/Sh1^* mice.

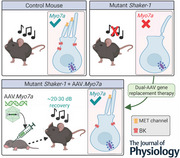

**Key points:**

- *Shaker‐1* mutant mice (*Myo7a^Sh1/Sh1^*), which carry a mutation in the unconventional myosin MYO7A and are the murine model of Usher 1B syndrome, become profoundly deaf at 1 month of age or soon after.
- In the mammalian cochlea, MYO7A is expressed in the hair cells, including within their actin‐rich stereociliary bundles.
- We show that hair cells of *Myo7a^Sh1/Sh1^* mice progressively lose their transducing stereocilia and mechanoelectrical transduction, and fail to acquire their fully mature basolateral current profile.
- Delivering exogenous *Myo7a* to newborn *Myo7a^Sh1/Sh1^* mice using dual‐adeno‐associated virus (AAVs) led to a substantial rescue of the bundle structure and function of inner hair cells, including mechanoelectrical transduction.
- This functional rescue led to a 20–30 dB improvement in hearing thresholds across most frequencies.
- These results support dual AAV‐induced gene replacement therapy as an effective strategy to recover the hair‐cell function in *Myo7a^Sh1/Sh1 ^* mice.

## Graphical abstract

**Abstract figure legend** *Shaker‐1* mice, a murine model of Usher 1B syndrome, carry a mutation in the unconventional myosin MYO7A. This results in the progressive loss of the two shortest rows of stereocilia that house the mechanoelectrical transducer (MET) channels in hair cells, leading to deafness shortly after hearing onset. Exogenous delivery of *Myo7a* to newborn *Shaker‐1* mice using dual‐adeno‐associated virus (AAV) vectors substantially rescued inner hair cell stereocilia and mechanoelectrical transduction, resulting in improved hearing function. BK: large conductance Ca^2+^ activated K^+^ channel.

## Introduction

*MYO7A* was the first deafness gene identified in humans causing both syndromic (*USHER 1B*) and non‐syndromic recessive deafness (Liu et al., 1997; Weil et al., 1995, 1997). Mice homozygous for *Myo7a* mutations exhibit the characteristic *shaker‐1* phenotype, which includes deafness, hyperactivity, head‐tossing and circling (Gibson et al., 1995; Lord & Gates, 1929). *Myo7a* encodes the unconventional myosin MYO7A, which is part of a class of motor proteins that couple ATP hydrolysis with mechanical force, allowing them to move along actin‐based filaments (Goode et al., 2000). In the mammalian cochlea, MYO7A is expressed in the hair cells, including within their stereociliary bundles (e.g. Hasson et al., 1995; Kolla et al., 2020; Rzadzinska et al., 2004; Scheffer et al., 2015; Underhill et al., 2025). Stereocilia are actin‐rich microvilli‐like structures required for the transduction of acoustic stimuli into a sensory receptor potential (Fettiplace & Kim, 2014). MYO7A has been implicated in several mechanisms, most of which are associated with the development of the hair bundles (Ballesteros et al., 2022; Moreland & Bird, 2022; Peng et al., 2009; Rzadzinska et al., 2009; Senften et al., 2006). More recently, MYO7A has been shown to be essential for maintaining the structural integrity of the mature hair bundles in post‐hearing mice (Underhill et al., 2025).

Several recessive mutations have been identified in *Myo7a*, all of which cause defects in the cochlear hair cells and lead to deafness, though the onset and progression of the dysfunction vary (Libby & Steel, 2001; Lord & Gates, 1929; Self et al., 1998). Mutation in the *Myo7a^4626SB^* allele results in a stop codon within the head domain of MYO7A, resulting in the absence of protein expression in the cochlea and severe hair cell abnormalities (Hasson et al., 1997; Kros et al., 2002; Mburu et al., 1997). The *Myo7a^816SB^* mutation, which results in a 10‐amino‐acid deletion in the MYO7A motor head core (Gibson et al., 1995; Mburu et al., 1997), also causes severe hair bundle abnormalities from early stages of development and profound deafness. The *Myo7a^6J^* mutation is an arginine to proline missense mutation that is also located in the core of the motor domain and is associated with a rapid progression of stereocilia dysfunction and deafness. Both *Myo7a^816SB^* and *Myo7a^6J^* mutations were predicted to have severe effects on protein stability (80–95% reduction: Hasson et al., 1997) and function (Self et al., 1998). In contrast to the mutations described above, the original *shaker‐1* mutant mice (*Myo7a^Sh1/Sh1^*), which also have an arginine‐to‐proline missense mutation located in a poorly conserved surface loop of the motor head (Gibson et al., 1995; Mburu et al., 1997), exhibit normal MYO7A expression in the hair cells. The hair bundles in these mice appear to develop normally, at least initially, with residual hearing function in very young adult mice (Self et al., 1998; Shnerson et al., 1983).

Adeno‐associated virus (AAV)‐mediated gene replacement therapy has successfully recovered hearing in mouse models with mutations in several genes required for hair cell function, including *Otof* (Akil et al., 2019; Al‐Moyed et al., 2019), *Tmc1* (Askew et al., 2015; Nist‐Lund et al., 2019) and *Vglut3* (Akil et al., 2012; Zhao et al., 2022). However, recovering cochlear function through AAV‐based replacement therapy has proven more challenging for genes that are critical during early stages of cochlear development (Amariutei et al., 2023). Indeed, a recent study aimed at reverting the cochlear phenotype in *Myo7a^4626SB/4626SB^* mice, characterized by severe hair bundle defects from early ages, was unable to rescue the hearing phenotype using dual‐AAV vector‐mediated expression of MYO7A in the inner ear (Lau et al., 2023). In the present study, we use *shaker‐1* mice (*Myo7a^Sh1/Sh1^*), which carry a mutation orthologous to that causing *USH1B* in humans (Weil et al., 1995), to test the capability of the dual‐AAV to rescue hair cell function and hearing. We show that the exogenous delivery of *Myo7a* using AAV8 or AAV9‐PhP.eB vectors was able to rescue several morphological and functional properties of IHCs, leading to an improvement in hearing thresholds of about 30 dB across most tested frequencies.

## Methods

### Ethical approval

Animal experimental work was licensed by the UK Home Office under the Animals (Scientific Procedures) Act 1986 (PCC8E5E93 and PP1481074) and was approved by the University of Sheffield Ethical Review Committee (180 626_Mar). Mice had free access to food and water and were on a 12 h light/dark cycle. Experiments were performed using mice carrying the spontaneous original *shaker‐1* mutation (*Myo7a^Sh1^*), obtained from the MRC Mary Lyon Centre (Harwell Campus, UK) and maintained on the original background (85% CBA and 15% mixed) (Gibson et al., 1995). Littermate mice of either sex were used for experiments.

For *ex vivo* experiments mice were killed by cervical dislocation followed by decapitation. For *in vivo* measurement of auditory brainstem responses (ABRs), mice were anaesthetized using intraperitoneal injection of ketamine (100 mg/kg body weight, Fort Dodge Animal Health, Fort Dodge, IA, USA) and xylazine (10 mg/kg, Rompun 2%, Bayer HealthCare LLC, Whippany, NY, USA). Mice were placed in a soundproof chamber for *in vivo* experiments following the loss of the retraction reflex with a toe pinch. After ABR recordings were carried out, mice were either killed by cervical dislocation or recovered with an intraperitoneal injection of atipamezole (1 mg/kg), permitting recovery from anaesthesia. For *in vivo* gene delivery, mice were anaesthetized with isoflurane (2.5%) delivered in 100% oxygen at a flow rate of 0.8 L/min. Mice under recovery from anaesthesia were returned to their cage, placed on a thermal mat, and monitored over the following 2–5 h until normal behaviour resumed.

All animal experiments performed in this study comply with *The Journal of Physiology*’s policies regarding animal experiments (https://physoc.onlinelibrary.wiley.com/hub/animal‐experiments).

### Tissue preparation

Both male and female mice were used for *ex vivo* experiments. Following cervical dislocation, the cochlea was dissected out and placed in a chamber containing an extracellular solution composed of (in mM): 135 NaCl, 5.8 KCl, 1.3 CaCl_2_, 0.9 MgCl_2_, 0.7 NaH_2_PO_4_, 5.6 d‐glucose and 10 Hepes‐NaOH. Amino acids, vitamins and sodium pyruvate (2 mm) were added from concentrates (Thermo Fisher Scientific, UK). The final pH was 7.48 and osmolality ∼308 mOsm/kg. Following the dissection, the apical coil of the cochlear sensory epithelium was transferred to a microscope chamber and immobilized via a nylon mesh attached to a stainless‐steel ring. The epithelia were continuously perfused with the above extracellular solution using a peristaltic pump (Cole‐Palmer, UK) connected to the microscope chamber, which was then mounted on the stage of an upright microscope (Olympus BX51, Tokyo, Japan; Leica DMLFS, Wetzlar, Germany). Microscopes were equipped with Nomarski differential interference contrast (DIC) optics, either 60× or 64× water immersion objective and 15× eyepieces.

### Auditory brainstem responses

Anaesthetized male or female mice were placed on a heated mat (37°C) inside a soundproof chamber (MAC‐3 acoustic chamber, IAC Acoustic, Chandler's Ford, UK). The mouse's pinna was positioned at 10 cm from the loudspeaker (MF1‐S, Multi Field Speaker, Tucker‐Davis Technologies, Alachua, FL, USA), which was calibrated daily with a low‐noise microphone probe system (ER10B+, Etymotic, USA). One subdermal electrode was positioned half‐way between the two pinna on the vertex of the cranium (active electrode) and the other two electrodes were placed under the skin behind the pinna of each ear (reference and ground electrode) as previously described (Carlton et al., 2024). Experiments were performed using customized software (Ingham et al., 2011) driving an RZ6 auditory processor (Tucker‐Davis Technologies). Response thresholds were estimated from the resulting ABR waveform, defined as the lower sound level at which any recognizable feature of the waveform was visible. Responses were recorded for pure tones of frequencies at 3, 6, 12, 18, 24, 30 and 36 kHz as well as broadband white noise clicks. Stimulus sound pressure levels were up to 95 or 120 dB SPL, presented in steps of 5 dB SPL (average of 256 repetitions). Tone bursts were 5 ms in duration with a 1 ms on/off ramp time presented at a rate of 42.6/s.

### Whole‐cell electrophysiology

Patch clamp recordings were performed at room temperature (20–24°C) using an Optopatch amplifier (Cairn Research Ltd, Faversham, UK) as previously described (Carlton et al., 2023). Patch pipettes were pulled from soda glass capillaries, which had a typical resistance in extracellular solution of 2–3 MΩ. The patch pipette intracellular solution contained (in mM): 131 KCl, 3 MgCl_2_, 1 EGTA‐KOH, 5 Na_2_ATP, 5 Hepes‐KOH, 10 Na‐phosphocreatine (final pH: 7.28; 294 mOsm/kg). Data acquisition was controlled by pClamp software using a Digidata 1440A (Molecular Devices, Sunnyvale, CA, USA). To reduce the electrode capacitance, patch electrodes were coated with surf wax (Mr Zoggs SexWax, USA). Recordings were low‐pass filtered at 2.5 kHz (8‐pole Bessel), sampled at 5 kHz and stored on a computer for off‐line analysis (Clampfit, Molecular Devices; Origin 2023: OriginLab, Northampton, MA, USA). Membrane potentials under voltage‐clamp conditions were corrected off‐line for the residual series resistance *R*_s_, which was normally compensated for by 80%, and the liquid junction potential (LJP) of −4 mV, which was measured between electrode and bath solutions.

To investigate the biophysical characteristics of the mechanoelectrical transducer (MET) current, hair bundles were displaced using a fluid‐jet system from a glass pipette driven by a 25 mm diameter piezoelectric disc (Carlton et al., 2021; Corns et al., 2014; Underhill et al., 2025). The fluid jet pipette tip was positioned near the hair bundles to elicit a maximal MET and contained the same extracellular solution mentioned above. Mechanical stimuli were applied as 50 Hz sinusoids (filtered at 1 kHz, 8‐pole Bessel). Prior to positioning of the fluid jet near the hair bundles, any steady‐state pressure was removed by monitoring the movement of debris in front of the pipette.

### AAV production

Due to size constrains, the full‐length coding sequence of the murine *Myo7a* cDNA (NM_0 011 00395.1) was split into two fragments: a 5′ fragment covering nucleotides 1–3108 and a 3′ fragment covering nucleotides 3109–6648. The 5′ construct (p0101_NterMYO7a) included the 5′ part of the *Myo7a* cDNA (encoding amino acids 1–1036) as well as a splice donor (SD) site. The 3′ construct (p0101_CterMyo7a) encompassed the 3′ part of the *Myo7a* cDNA (encoding amino acids 1037–2215) and a splice acceptor (SA) site. The SA and SD sites enabled the reconstruction of the full‐length transcript via trans‐splicing within the transduced cells. All these fragments were synthesized by GenScript (Piscataway, NJ, USA). Both constructs, sharing the alkaline phosphatase recombinogenic bridging sequence (AP), were inserted into a modified version of the pAAV.CMV.PI.EGFP.WPRE.bGH vector plasmid (Addgene, Cambridge, MA, USA), in which the EGFP coding sequence was replaced by the *Myo7a* fragment. This resulted in the generation of a pair of recombinant vectors referred to as AAV‐MYO7A‐N‐term and AAV‐MYO7A‐C‐term. The recombinant vectors were packaged in‐house into the AAV2/8 or AAV9‐PhP.eB capsids. AAV titres were expressed in viral genomes per millilitre (vg/mL) as determined by a fluorometric assay: AAV8‐MYO7A‐N‐term (1.0 × 10^13^ vg/mL); AAV8‐MYO7A‐C‐term (6.3 × 10^12^ vg/mL); AAV9‐PhP.eB‐MYO7A‐N‐term (1.5 × 10^13^ vg/mL); AAV9‐PhP.eB‐MYO7A‐C‐term (6.8 × 10^12^ vg/mL); AAV8‐GFP (2.8 × 10^13^ vg/mL); AAV9‐PhP.eB‐GFP (2.2 × 10^13^ vg/mL). Final concentrated AAV vector stocks were stored in phosphate‐buffered saline (PBS) with MgCl_2_ (1 mM) and KCl (2.5 mM) at −70°C.

### AAV gene delivery in mice

The surgical protocol used for AAV injection into the cochlea of P0–P1 *Myo7a^Sh1^* mice was performed under general anaesthesia. The left or right ear was accessed via an incision just below the pinna as previously described (Jeng et al., 2022; O'Connor et al., 2024). When the round window membrane (RWM) was identified, it was gently punctured with a borosilicate pipette. This was followed by the injection of the AAV into the cochlea (pressure controlled by mouth) of 1 or 2 µL of the AAVs. For injections involving dual‐AAV constructs, AAV8‐*Myo7a* (1.2 × 10^12^ vg/mL) or AAV9‐PhP.eB‐*Myo7a* (6.8 × 10^12^ vg/mL) were used, prepared by combining equal volumes of the corresponding N‐terminal and C‐terminal vectors. Following the injection, the pipette was retracted from the RWM and the wound was closed with veterinarian glue.

### Scanning electron microscopy

After dissecting out the inner ear from the mouse, the cochlea was gently perfused with fixative for 1—2 min through the round window using a 10 µL pipette tip. A small hole in the apical portion of cochlear bone was made prior to perfusion to allow the fixative to flow out from the cochlea. The fixative contained 2.5% (v/v) glutaraldehyde in 0.1 M sodium cacodylate buffer plus 2 mm CaCl_2_ (pH 7.4). The inner ears were then immersed in the above fixative and placed on a rotating shaker for 2 h at room temperature. After the fixation, the cochleae were washed in the same cacodylate buffer and the organ of Corti was exposed by removing the bone from the apical coil of the cochlea. Samples were then immersed in 1% osmium tetroxide in 0.1 m cacodylate buffer for 1 h. For osmium impregnation, which avoids gold coating, cochleae were incubated in solutions of saturated aqueous thiocarbohydrazide (20 min) alternating with 1% osmium tetroxide in buffer (2 h) twice (the OTOTO technique: Furness & Hackney, 1986). The cochleae were then dehydrated through an ethanol series and critical point dried using CO_2_ as the transitional fluid (Leica EM CPD300) and mounted on specimen stubs using conductive silver paint (Agar Scientific, Stansted, UK). The apical coil of the sensory epithelium was examined at 10 kV using a Tescan Vega3 LMU scanning electron microscope (Cryo‐Electron Microscopy facility, University of Sheffield) or an FEI Inspect F scanning electron microscope (Sorby Centre for Electron Microscopy, University of Sheffield). At least three mice were processed for each genotype. Images were taken from the same region (around 12 kHz) used for the electrophysiological recordings.

### Immunofluorescence microscopy

For pre‐hearing mice, the inner ear was dissected out and immersed for 20 min at room temperature in a solution containing 4% paraformaldehyde in PBS (pH 7.4). For adult mice, the inner ear was initially gently perfused with the above solution for 1–2 min through the round window prior to the 20 min fixation described for pre‐hearing mice. Following fixation, the inner ears were then washed three times in PBS for 10 min and the sensory epithelia dissected out using fine forceps and incubated in PBS supplemented with 5% normal goat or horse serum and 0.5% Triton X‐100 for 1 h at room temperature. The samples were immunolabelled with primary antibodies overnight at 37°C, washed three times with PBS and incubated with the secondary antibodies for 1 h at 37°C. Antibodies were prepared in 1% serum and 0.5% Triton X‐100 in PBS. Primary antibodies were as follows: rabbit IgG anti‐myosin 7a (1:500, Proteus Biosciences, #25‐6790; Ramona, CA, USA), mouse IgG1 anti‐BK (1:500, Antibodies Incorporated, 75–408; Davis, CA, USA), mouse IgG2a anti‐GluR2 (1:200, Millipore, MAB397; Billerica, MA, USA) and mouse IgG1 anti‐CtBP2 (1:500, BD, 612 044; Franklin Lakes, NJ, USA). F‐actin was stained with Texas Red‐X phalloidin (1:1000, ThermoFisher, T7471;.Waltham, MA, USA) within the secondary antibody solution. Secondary antibodies were species appropriate Alexa Fluor or Northern Lights. Samples were mounted in Vectashield (H‐1000). The images from the apical cochlear region (around 12 kHz) were captured with Nikon A1 or ZEISS LSM980 Airyscan confocal microscopes (Wolfson Light Microscope Facility at the University of Sheffield). Image stacks were processed with Fiji ImageJ software. At least three mice for each genotype were used for each experiment.

### Statistical analysis

Statistical comparisons were made by Student's *t* test or Mann–Whitney *U* test (when a normal distribution could not be assumed). For multiple comparisons, one‐way ANOVA followed by a suitable post test was used for normally distributed data, otherwise Kruskal–Wallis with Dunn's post test was used. *P *< 0.05 was selected as the criterion for statistical significance. Average values are quoted in the text and figures as means ± SD Animals of either sex were randomly assigned to the different experimental groups. No statistical methods were used to define sample size, which was defined based on similar previously published work from our laboratory. Animals were taken from several cages and breeding pairs over a period of several months.

## Results

### Missense mutation in the *shaker‐1* gene disrupts the hair bundle morphology and causes hearing loss

**Figure 1:**
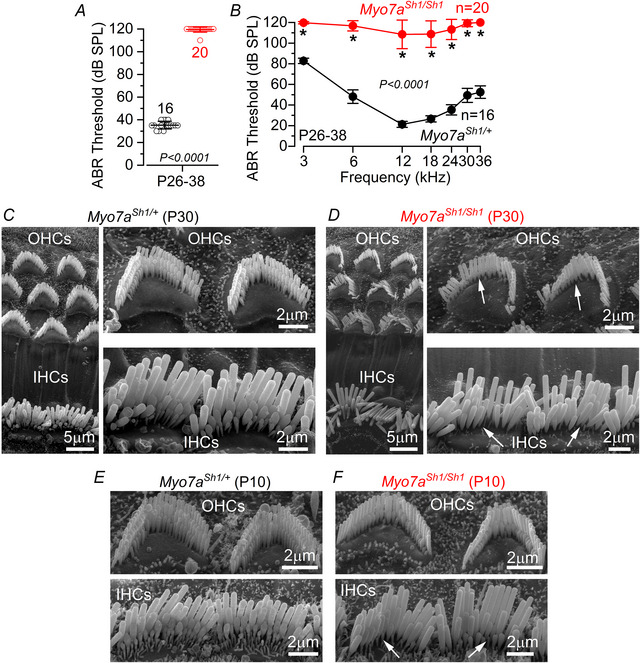
Hearing function and hair bundle morphology in *Myo7a^Sh1^* mice *A*, average auditory brainstem response (ABR) thresholds for click stimuli recorded from control *Myo7a^Sh1/+^* (black) and *Myo7a^Sh1/Sh1^* (red) mice at postnatal day 26–38 (P26–38). Click thresholds were significantly elevated in *Myo7a^Sh1/Sh1^* compared to control mice (*P* < 0.0001 Mann–Whitney *U*‐test). *B*, ABR thresholds for frequency‐specific pure tone burst stimuli at 3, 6, 12, 18, 24, 30 and 36 kHz recorded from controls and littermate *Myo7a^Sh1/Sh1^* mice at P26–38 (^*^ *P* < 0.0001 from Šidák's post test, two‐way ANOVA). In both *A* and *B*, the numbers of mice tested for each genotype are shown next to the symbols and values are reported as mean ± SD. *C*–*F*, example of scanning electron microscope images showing the hair bundles of IHCs and OHCs from the apical coil of the cochlea at P30 (*C* and *D*) and P10 (*E* and *F*) of control (*C* and *E*) and *Myo7a^Sh1/Sh1^* (*D* and *F*) mice. Arrows point to the missing stereocilia in the second and/or third row of the hair cells from *Myo7a^Sh1/Sh1^* mice.

ABRs, which reflect the electrical activity of the afferent spiral ganglion neurons and downstream auditory pathway, were used to investigate the hearing function of *Myo7a^Sh1^* mice. ABR thresholds for click sound stimuli were absent at P26–P38 in *Myo7a^Sh1/Sh1^* compared to *Myo7a^Sh1/+^* mice (*P* < 0.0001, Fig. 1*A*). When frequency‐specific pure tone burst stimuli were used, ABR thresholds were also absent or highly elevated in *Myo7a^Sh1/Sh1^* compared to control (*Myo7a^Sh1/+^*) mice (*P* < 0.0001, two‐way ANOVA, Fig. 1*B*). Hearing loss in *Myo7a^Sh1/Sh1^* mice was associated with the lack of the shorter second and third rows of stereocilia in both inner hair cells (IHCs) and outer hair cells (OHCs) (Fig. 1*C* and *D*). Since the shorter stereocilia rows are those with the MET channels at their tip (Beurg et al., 2009), the loss of channels is likely to be the primary cause of deafness in *shaker‐1* mice. Although previous studies have indicated that the hair bundles of both hair cell types develop normally, at least during early postnatal ages (Kros et al., 2002; Self et al., 1998), we found that the loss of stereocilia was already evident at P10 (Fig. 1*E* and *F*), which is before the onset of hearing (about P12–P13: Shnerson & Pujol, 1982).

### The basolateral membrane profile of OHCs and IHCs in *Myo7a^Sh1^* mice

Since a functional MET apparatus has been shown to be essential for maintaining the biophysical identity of adult IHCs and OHCs (Corns et al., 2018; O'Connor et al., 2024), we investigated whether a similar functional regulation was also present in *Myo7a^Sh1/Sh1^* mice that lack the transducing stereocilia. Basolateral membrane currents were recorded from immature and mature hair cells by applying a series of voltage steps from −124 mV to more positive potentials in 10 mV nominal increments from the holding potential of −84 mV.

**Figure 2:**
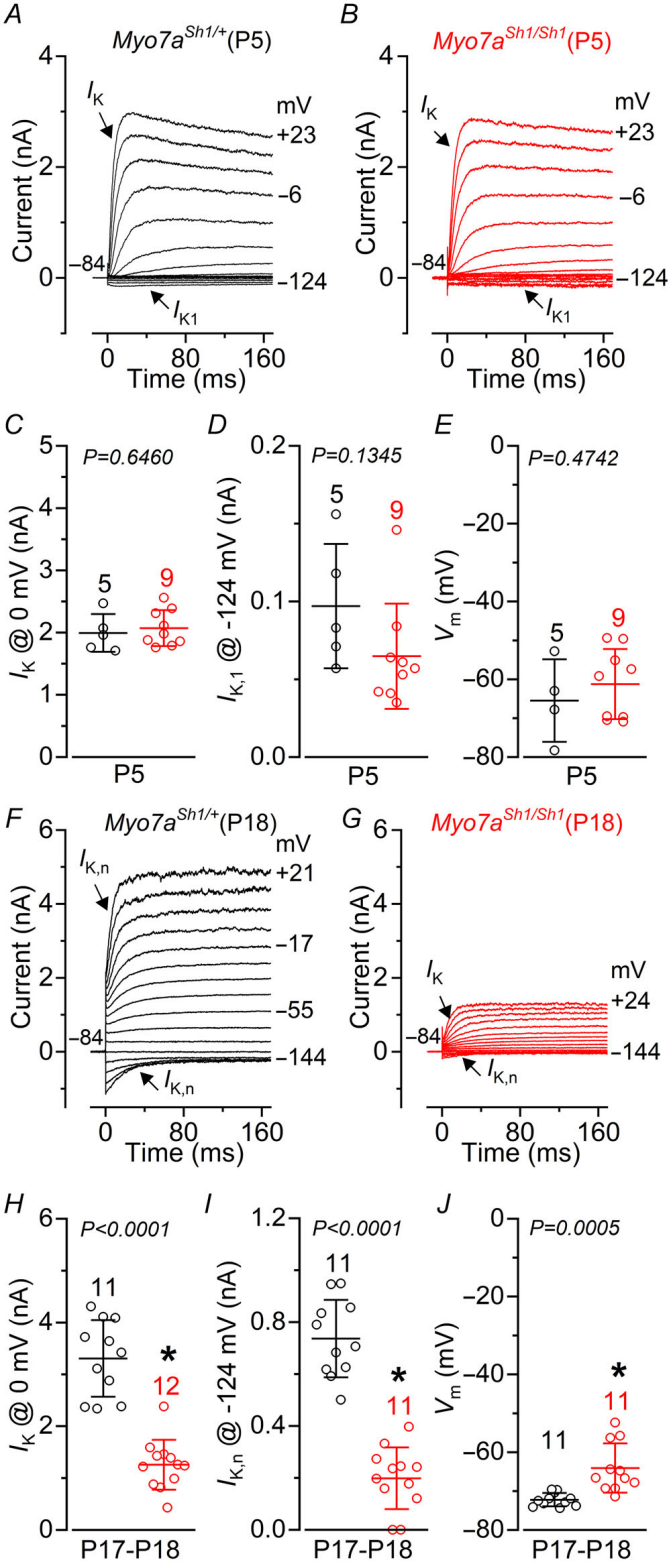
OHCs from *Myo7a^Sh1/Sh1^* mice fail to develop their characteristic mature current profile *A* and *B*, current responses from apical coil OHCs of immature P5 control *Myo7a^Sh1/+^* (*A*) and *Myo7a^Sh1/Sh1^* (*B*) mice. Current recordings were elicited by using depolarizing and hyperpolarizing voltage steps (10 mV increments) from the holding potential of –84 mV to the various test potentials shown by some of the traces. Note the similar size and time‐course of the outward and inward K^+^ currents in both genotypes. *C* and *D*, average size of the peak total outward K^+^ current measured at 0 mV *I*_K_ (*C*) and *I*_K,1_ measured at –124 mV (*D*) in OHCs from control (black) and *Myo7a^Sh1/Sh1^* (red) mice. *E*, average resting membrane potential measured in the OHCs from both genotypes. *F* and *G*, current responses from mature P18 OHCs of control (*F*) and *Myo7a^Sh1/Sh1^* (*G*) mice obtained as described in *A* and *B. H* and *I*, average size of the peak total outward K^+^ current measured at 0 mV *I*_K_ (*H*) and *I*_K,n_, which was measured as the difference between the peak and steady‐state of the deactivating inward current at –124 mV (*I*) in OHCs from control (black) and *Myo7a^Sh1/Sh1^* (red) mice. *J*, average resting membrane potential measured in the OHCs from both genotypes. Data in *C*–*E* and *H*–*J* are plotted as mean ± SD. Single cell value recordings (open symbols) are plotted behind the average bars. Statistical tests shown are obtained using a *t* test. Number of IHCs investigated is shown above the average data points.

In immature P5 OHCs, we found that their resting membrane potential and current profile, which is defined by the expression of a delayed outward rectifying K^+^ current (*I*_K_) and an inward rectifying K^+^ current (*I*_K1_), was indistinguishable between control and *Myo7a^Sh1/Sh1^* mice (Fig. 2*A*–*E*). This indicates that the initial development of the MET apparatus is likely to be unaffected by the *shaker‐1* mutation, as previously suggested (Self et al., 1998). The onset of functional maturation in apical OHCs occurs at around P7–P8 marked by the downregulation of ion channels that carry the immature‐type currents (e.g. P5: Fig. 2*A* and *B*), the emergence of the negatively activating K^+^ current *I*_K,n_ carried by KCNQ4 channels (Kubisch et al., 1999) required for setting the resting membrane potential (Marcotti & Kros, 1999) and the acquisition of electromotility (Abe et al., 2007). Although mature OHCs from P17–P18 *Myo7a^Sh1/Sh1^* mice successfully down‐regulated their immature‐type currents, they failed to fully upregulate *I*_K,n_ (Fig. 2*F*–*I*), resulting in a significantly more depolarized resting membrane potential compared to control cells (Fig. 2*J*). These data suggest that a defect in the MET apparatus of OHCs from *Myo7a^Sh1/Sh1^* mice is likely to occur at or following the onset of maturation during the second postnatal week.

**Figure 3:**
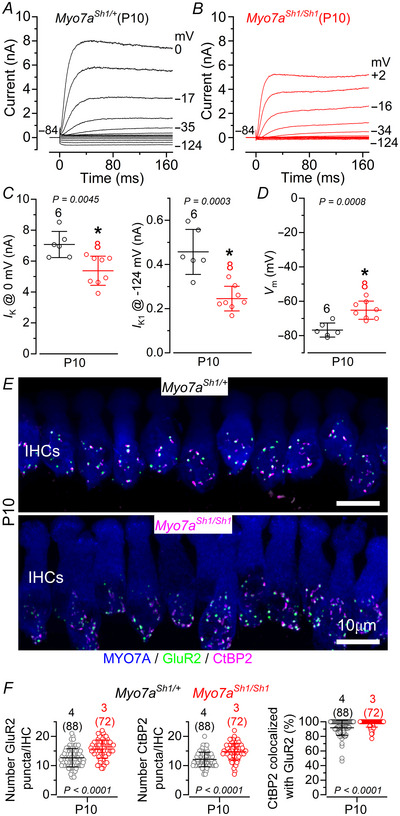
*Shaker‐1* mutation affects the IHC membrane current of both pre‐ and post‐hearing ages *A* and *B*, current responses from IHCs of control *Myo7a^Sh1/+^* (*A*) and *Myo7a^Sh1/Sh1^* (*B*) P10 mice. Current recordings were elicited as described in Fig. 1. *C* and *D*, average size of the characteristic K^+^ currents present in immature IHCs: outward delayed rectifier *I*_K_, which was measured at 0 mV (*C*) (Marcotti et al., 2003), and the inward rectifier *I*_K,1_, which was measured at –124 mV(*D*) (Marcotti et al., 1999) from control (black) and *Myo7a^Sh1/Sh1^* (red) P1 mice. Data in *C* and *D* are plotted as mean ± SD. Single cell value recordings (open symbols) are plotted behind the average data. Statistical tests shown are obtained using a *t* test. Number of IHCs investigated is shown above the average data points. *E*, maximum intensity projections of confocal z‐stacks of IHCs taken from the apical cochlear region of *Myo7a^Sh1/+^* (upper panel) and *Myo7a^Sh1/Sh1^* (lower panel) P10 mice. Cochleae were immunostained using antibodies against CtBP2 (ribbon synaptic marker: magenta) and GluR2 (postsynaptic marker: green). Myosin 7a (MYO7A) was used as the IHC marker (blue). *F*, number of GluR2 (left panel), CtBP2 (middle) and colocalized GluR2‐CtBP2 puncta (right) from *Myo7a^Sh1/+^* and *Myo7a^Sh1/Sh1^* P10 mice. Statistical analysis was performed using the Mann–Whitney *U‐*test. Data are plotted as mean values and individual counts (open symbols) are also shown. Data are plotted as mean ± SD.

IHC functional maturation occurs at around the onset of hearing (P12: Kros et al., 1998; Marcotti et al., 2003), by which time OHCs have already acquired mature‐like characteristics (Marcotti & Kros, 1999). We found that the size of both *I*_K_ and *I*_K1_ in IHCs was already significantly reduced in P10 *Myo7a^Sh1/Sh1^* mice compared to control littermates (Fig. 3*A*–*D*), a developmental stage when hair bundle defects are already apparent (Fig. 1*C* and *D*). A previous study showed that IHC ribbon synapses from mice lacking the MET current fail to develop normally, with their number appearing normal at P2 but becoming markedly elevated by P7, indicating defects in their developmental refinement (Lee et al. 2021). We made similar observations in the *shaker‐1* mutant, with the number of pre‐synaptic ribbons (CtBP2 puncta), post‐synaptic AMPA glutamate receptors (GluR2 puncta) and their colocalization being significantly increased in *Myo7a^Sh1/Sh1^* mice compared to control littermates (Fig. 3*E* and *F*).

**Figure 4:**
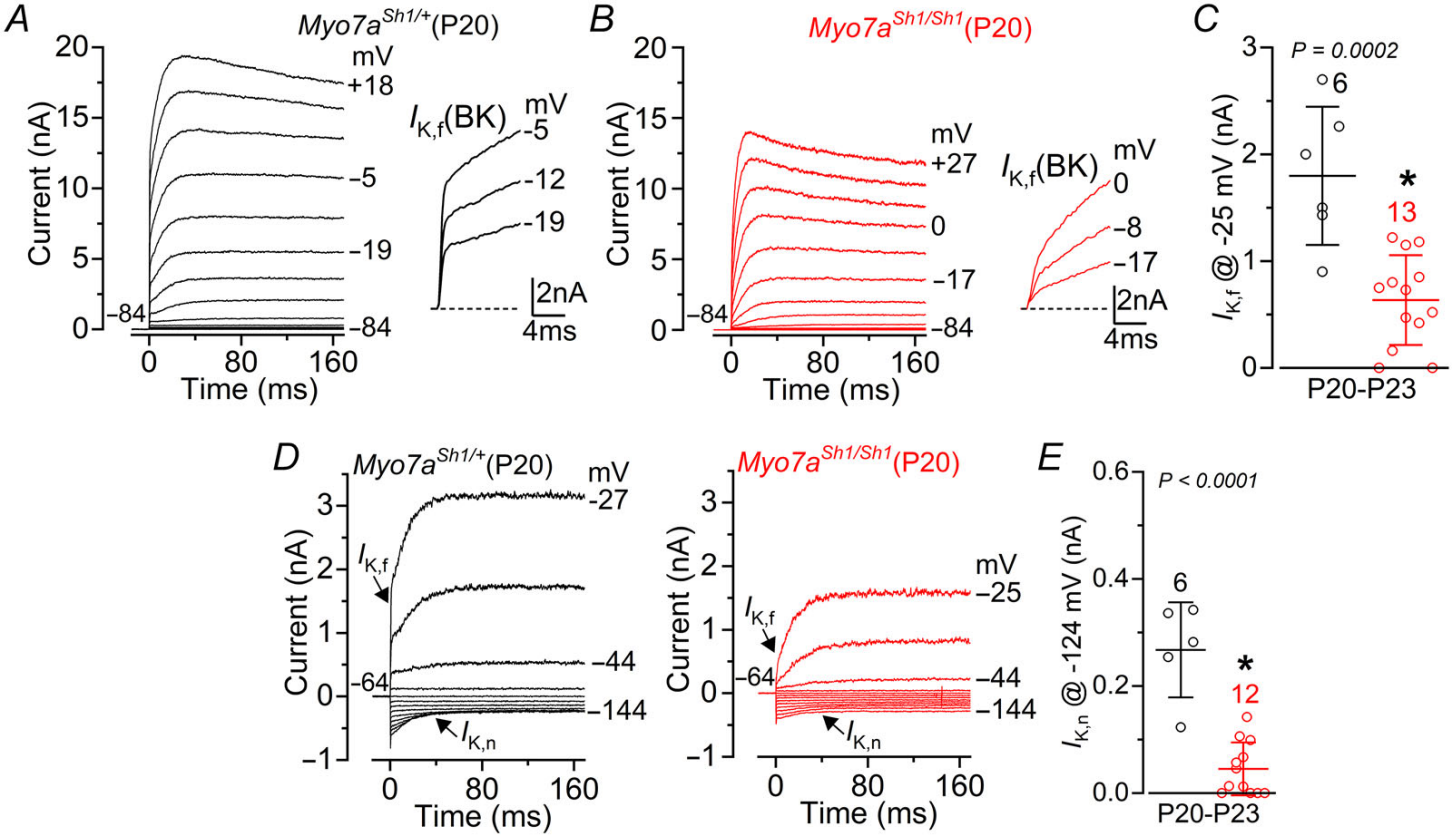
Mature IHCs from *shaker‐1* mice retain an immature‐like basolateral membrane current profile *A* and *B*, current responses from IHCs of control *Myo7a^Sh1/+^* (*A*) and *Myo7a^Sh1/Sh1^* (*B*) P20 mice. The fast activation of the BK current (*I*_K,f_) is better appreciated in the expanded time scale (see insets). *C*, the size of the outward K^+^ current *I*_K,f_, which was measured at –25 mV and at 1 ms from the onset of the voltage step (Marcotti et al., 2003). *D*, current responses from IHCs of control *Myo7a^Sh1/+^* (left) and *Myo7a^Sh1/Sh1^* (right) P20 mice, elicited by using hyperpolarizing and depolarizing voltage steps (10 mV increments) from the holding potential of –64 mV to the various test potentials shown by some of the traces. This protocol is used to emphasize the presence of *I*_K,n_. *E*, the size of *I*_K,n_, which was measured as the difference between the peak and steady state of the deactivating inward current at –124 mV from control (black) and *Myo7a^Sh1/Sh1^* (red) P20–P23 mice. Data in *C* and *E* are plotted as mean ± SD. Single cell value recordings (open symbols) are plotted behind the average data. Statistical tests shown are obtained using a *t* test. Number of IHCs investigated is shown above the average data points.

Mature IHCs express a rapidly activating, large conductance Ca^2+^‐activated K^+^ current carried by BK channels, named *I*_K,f_ (Kros et al., 1998; Lingle et al., 2019; Marcotti et al., 2004; Thurm et al., 2005), which was evident in control cells but largely reduced in *Myo7a^Sh1/Sh1^* mice (Fig. 4*A*–*C*). In addition, post‐hearing IHCs express, like OHCs, *I*_K,n_ (Kros et al., 1998; Marcotti et al., 2003; Oliver et al., 2003), which was also largely reduced in *Myo7a^Sh1/Sh1^* mice (Fig. 4*D* and *E*). These results highlight that the mutation in *Myo7a* present in *shaker‐1* mice disrupts the normal acquisition of the biophysical characteristics of mature hair cells.

### AAV‐mediated *Myo7a* replacement in new born mice improves hearing thresholds and repairs IHC morphological and biophysical defects in *Myo7a^Sh1^* mice

**Figure 5:**
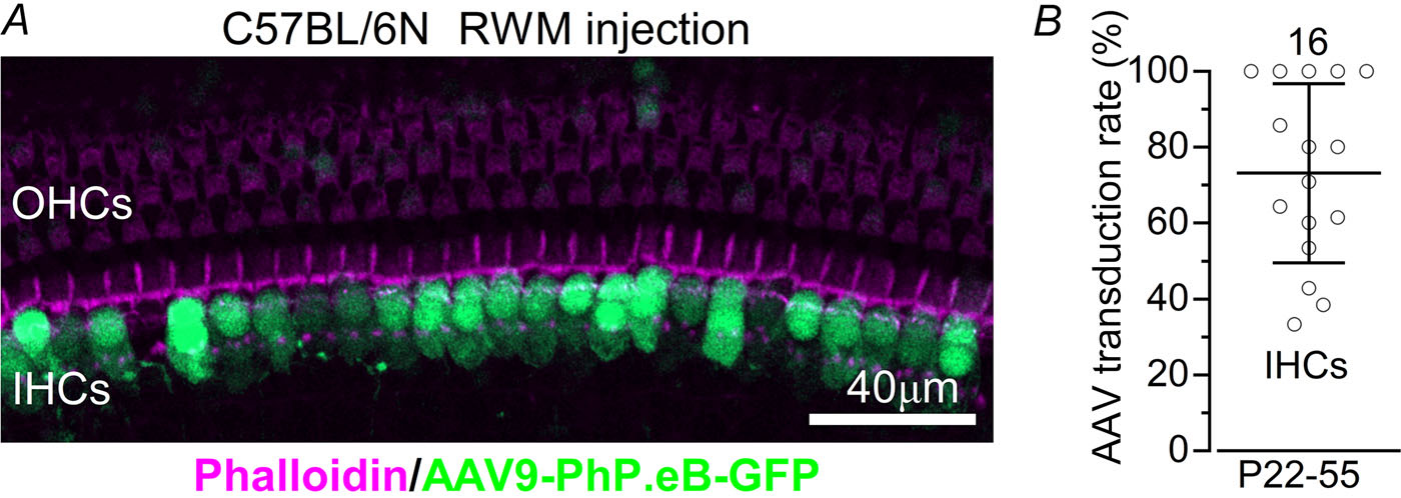
Hair cell transduction efficiency of AAVs *A*, confocal images obtained from the apical coil of the cochlea from wild‐type mice (C57BL/6N), which were transduced with AAVs‐*GFP* (AAV8‐*GFP* or AAV9‐PhP.eB‐*GFP*) through the round window membrane (RWM: see Methods) at P1–P3. Dissected cochleae were fixed, stained with Texas red phalloidin, and imaged for both phalloidin and GFP. While the large majority of IHCs were GFP positive, only a few OHCs expressed GFP. *B*, viral‐transduction rates in apical IHCs were determined from the number of GFP‐positive cells normalized by the total hair cells identified with phalloidin in the field of view. Data are plotted as mean ± SD.

To determine whether hearing loss caused by the missense mutation *Myo7a^Sh1^* can be reversed by AAV‐based gene replacement therapy, we generated dual‐AAV8‐*Myo7a* and dual‐AAV9‐PhP.eB‐*Myo7a* vectors. Since similar results were obtained with both AAV serotypes, the data were pooled for analysis. We first investigated whether both the AAV8 and AAV9‐PhP.eB serotypes were effective in transducing hair cells in the inner ear of the *shaker‐1* mice by assessing their transduction efficiency using GFP‐only reporter constructs. We injected AAV8‐GFP or AAV9‐PhP.eB‐GFP into the perilymphatic space via the RWM in P1–P3 C57BL/6N mice and quantified GFP‐positive hair cells (Fig. 5*A*). We quantified only the number of GFP‐positive IHCs (Fig. 5*B*), as OHCs were rarely transduced by either AAV vector (Fig. 5*A*).

**Figure 6:**
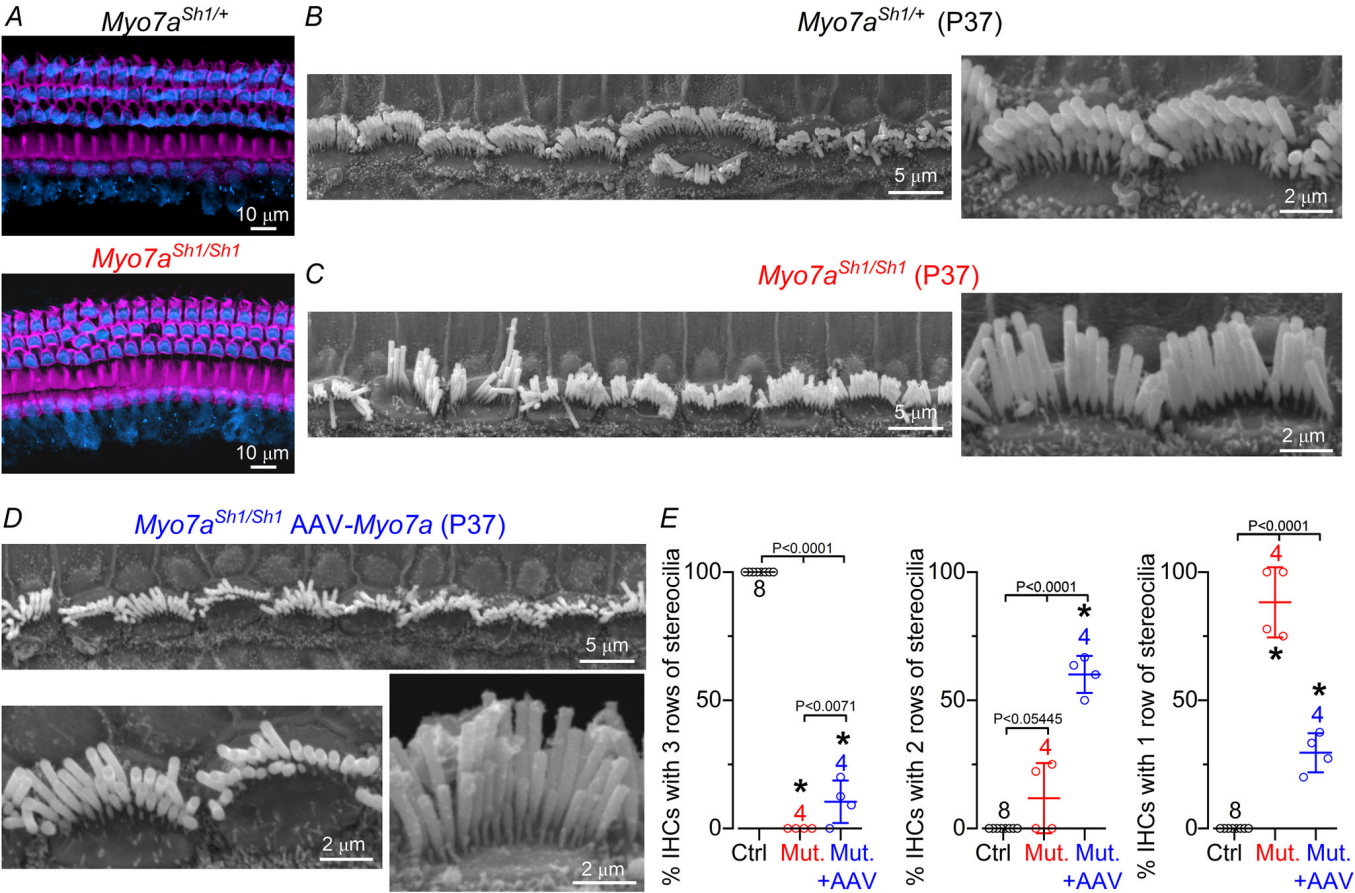
Improved hair bundle morphology in *Myo7a^Sh1/Sh1^* mice injected with AAV‐*Myo7a* *A*, maximum intensity projections of confocal z‐stack images taken from the apical region of the cochlea in non‐injected control (top) and *Myo7a^Sh1/Sh1^* (bottom) adult mice showing that MYO7A (blue) is expressed in all hair cells. The stereocilia actin core was labelled with phalloidin (magenta). *B*–*D*, scanning electron microscope images showing the IHC hair bundle structure in the apical coil region of the cochlea of P37 control *Myo7a^Sh1/+^* (*B*), *Myo7a^Sh1/Sh1^* (*C*) and *Myo7a^Sh1/Sh1^AAV‐Myo7a* (*D*) mice at different magnifications. *E*, average number of IHCs in the field of view (about 60 µm) showing three rows (left), two rows (middle) or one row (right) of stereocilia. Data are plotted as mean ± SD. Single cell value recordings (open symbols) are plotted behind the average bars. Statistical tests shown are obtained using Tukey's post test (one‐way ANOVA). Number of cochleae investigated is shown above the average data points.

Since the missense mutation in *Myo7a^Sh1^* does not prevent the expression of MYO7A in hair cells, it was not possible to identify which hair cells had been transduced by the AAV‐*Myo7a* (Fig. 6*A*). Considering that the main phenotype of the *shaker‐1* mutation is the loss of the two transducing rows of stereocilia (rows 1 and 2: see Fig. 1), we assessed the degree of IHC recovery by quantifying the number of rows in control, *Myo7a^Sh1/Sh1^* and *Myo7a^Sh1/Sh1^* mice injected with AAV‐*Myo7a* (Fig. 6*B*–*E*). While the hair bundles of P37 control IHCs were all formed by three rows of stereocilia (Fig. 6*B* and *E*), those from littermate *Myo7a^Sh1/Sh1^* mice consisted primarily of a single row of stereocilia (Fig. 6*C* and *E*). *Myo7a^Sh1/Sh1^* mice injected with AAV‐*Myo7a* showed a significantly increased number of IHCs with two and three rows of stereocilia compared to *Myo7a^Sh1/Sh1^* mice (Fig. 6*D* and *E*).

**Figure 7:**
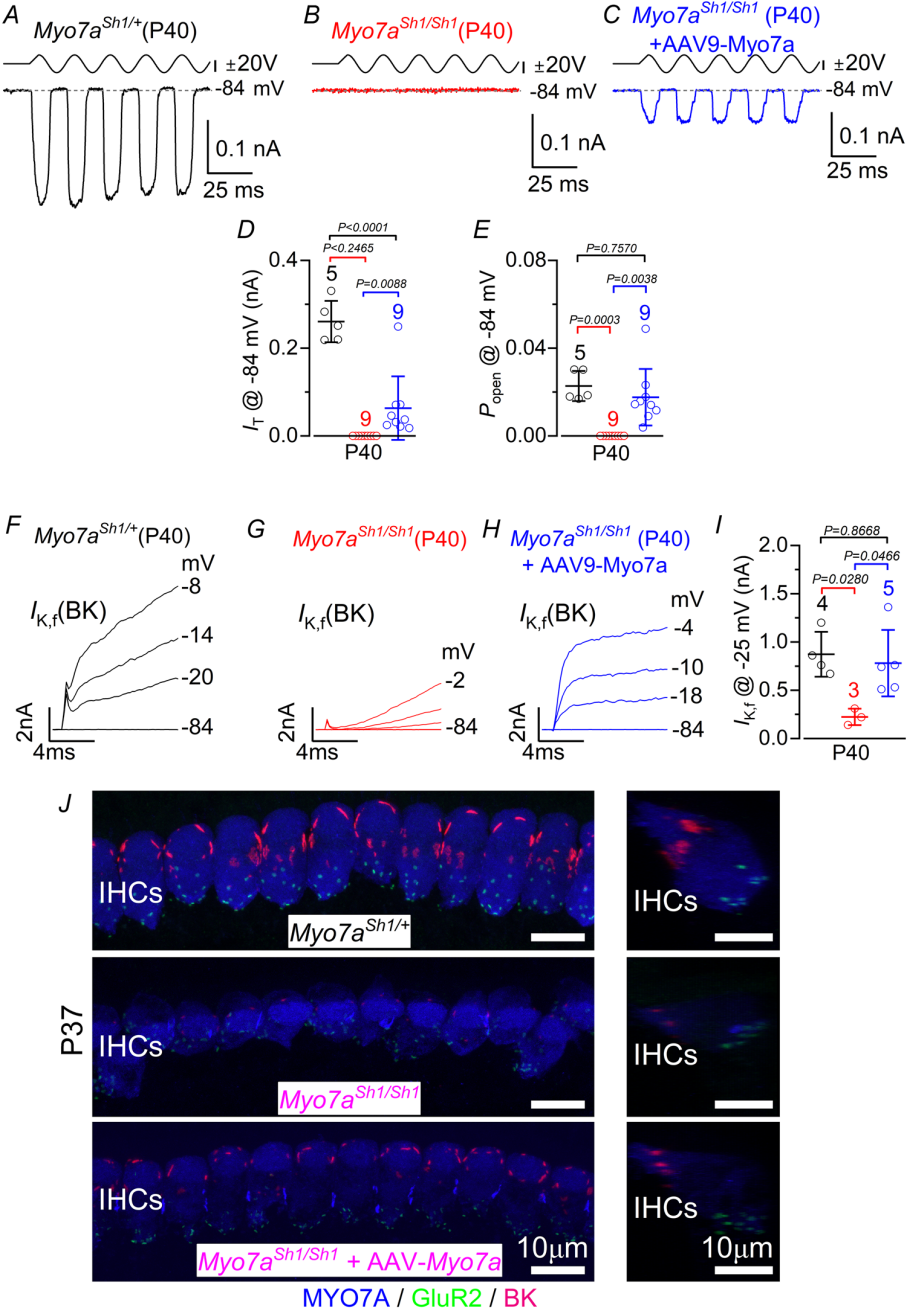
IHCs from *Myo7a^Sh1^* mice transduced with AAV‐*Myo7a* delivery recover mechanoelectrical transduction and basolateral membrane potassium currents *A*–*C*, saturating MET currents recorded from apical‐coil IHCs of P40 control (*A*), *Myo7a^Sh1/Sh1^* (*B*) and *Myo7a^Sh1/Sh1^AAV‐Myo7a* (*C*) mice. MET currents were elicited in response to 50 Hz sinusoidal force stimuli to the hair bundles at the membrane potential of –84 mV in the presence of 1 mm intracellular EGTA. Driver voltage (DV) stimuli to the fluid jet are shown above the traces (positive DV being excitatory). *D*, maximum size of the MET current recorded from P40 IHCs from control (black), *Myo7a^Sh1/Sh1^* (red) and *Myo7a^Sh1/Sh1^AAV‐Myo7a* (blue) mice at –84 mV. *E*, resting open probability (*P*_o_) of the MET channel in P40 IHCs from the three experimental conditions at –84 mV. *F*–*H*, activation time course of the BK current (*I*_K,f_) recorded from IHCs of control (*F*), *Myo7a^Sh1/Sh1^* (*G*) and *Myo7a^Sh1/Sh1^AAV‐Myo7a* (*H*) P40 mice. *I*, size of *I*_K,f_ measured at –25 mV and at 1 ms from the onset of the voltage step. Data in *D*, *E* and *I* are plotted as mean ± SD. Single cell value recordings (open symbols) are plotted behind the average bars. Statistical tests shown are obtained using Tukey's post test (one‐way ANOVA). Number of IHCs investigated is shown above the average data points. *J*, maximum intensity projections of confocal z‐stacks taken from the apical cochlear region of control (top), *Myo7a^Sh1/Sh1^* (middle) and *Myo7a^Sh1/Sh1^AAV8‐Myo7a* (bottom) P40 mice using antibodies against BK (red: located at the IHC neck region), the post‐synaptic marker GluR2 (green) and the hair cell marker MYO7A (blue). Right panels show maximum intensity projections of confocal z‐stack images of one IHC from the right panels but viewed from the side. Scale bars are 10 µm.

Considering the much‐improved hair bundle morphology following AAV‐*Myo7a* injection, we investigated whether this led to a recovery in the biophysical properties of IHCs. MET currents were investigated from mature IHCs in response to hair bundle displacement using a 50 Hz sinusoidal force stimulus from a piezo‐driven fluid jet (Carlton et al., 2021; Corns et al., 2018; Underhill et al., 2025). Hair bundle displacement in the excitatory direction (i.e. towards the taller stereocilia) elicited an inward MET current at the holding potential of −84 mV in control P40 mice (Fig. 7*A* and *D*). The same approach applied to the IHCs of *Myo7a^Sh1/Sh1^* mice failed to elicit any MET current (Fig. 7*B* and *D*), which agrees with most of these cells having lost the two transducing rows of stereocilia (Fig. 6). However, an MET current, although smaller than that in control IHCs, was present in *Myo7a^Sh1/Sh1^* mice injected with AAV‐*Myo7a* (Fig. 7*C* and *D*), consistent with most of them having regained one or both transducing rows of stereocilia (Fig. 6). The resting open probability (*P*_o_) of the MET channel, which is responsible for the current flowing in the absence of mechanical stimulation, was also present in *Myo7a^Sh1/Sh1^* mice injected with AAV‐*Myo7a*, the size of which was comparable to that of the IHCs from control mice (Fig. 7*E*). In addition, IHCs from *Myo7a^Sh1/Sh1^* mice injected with AAV‐*Myo7a* expressed the rapidly activating *I*_K,f_ to a level comparable to that recorded in the IHCs of control mice (Fig. 7*F*–*J*).

**Figure 8:**
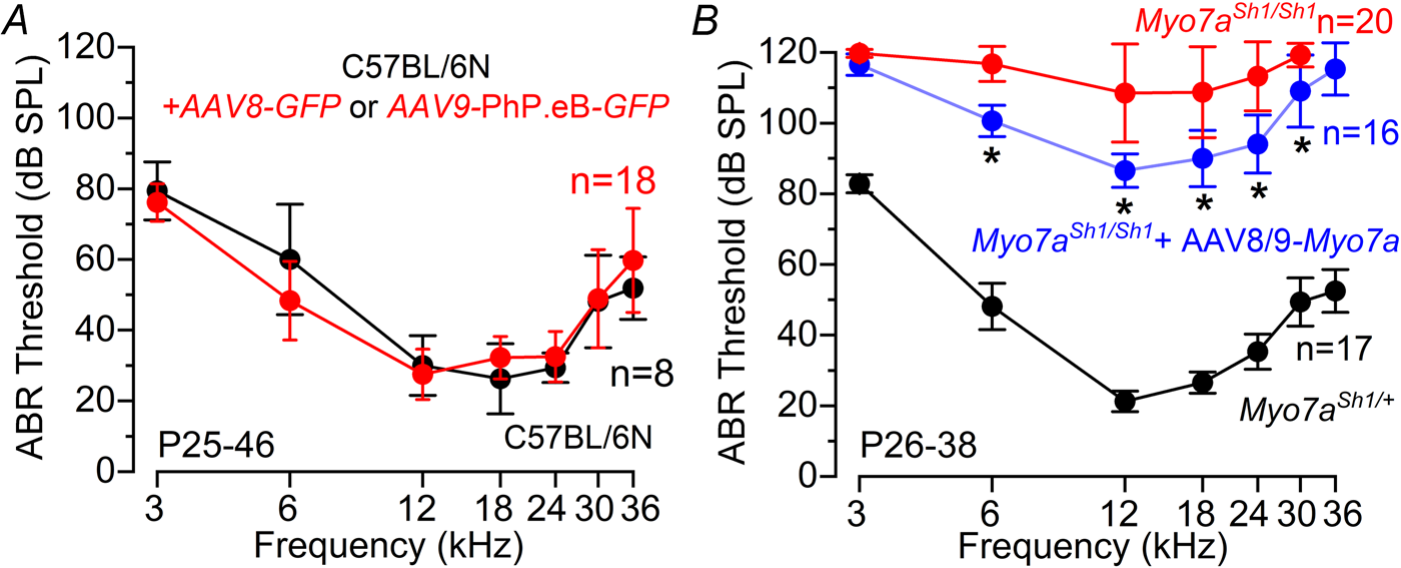
Improved ABR thresholds in *Myo7a^Sh1/Sh1^* mice injected with AAV‐*Myo7a* *A*, ABR thresholds for frequency‐specific pure tone stimulation between 3 and 36 kHz recorded from non‐injected (black) and injected (red: surgery and RWM injection of AAVs‐*GFP*) mice at P25–P46. *B*, average ABR thresholds for frequency‐specific pure tone burst stimuli recorded from control *Myo7a^Sh1/+^* (black), *Myo7a^Sh1/Sh1^* (red) and *Myo7a^Sh1/Sh1^AAVs‐Myo7a* (blue: AAV8‐*GFP* or AAV9‐PhP.eB‐*GFP*) mice at P26–38. The number of mice tested for each experimental conditions are shown next to the data (mean ± SD).

Hearing function in these mice was then investigated at P25–P46 with ABR recordings, as described in Fig. 1. We found that ABR thresholds in mice that underwent the surgical procedure and delivery of AAV‐GFP at P1–P3 were comparable to those of untreated control mice (*P* = 0.9805, two‐way ANOVA, Fig. 8*A*). We then delivered the dual AAV vector carrying *Myo7a* into the cochlea via the RWM of P0–P1 control *Myo7a^Sh1/+^* and *Myo7a^Sh1/Sh1^* mice. Functional recovery was assessed between P26 and P38. ABR thresholds in response to pure tone burst stimuli differed significantly across experimental groups (*P* < 0.0001, two‐way ANOVA, Fig. 8*B*). Notably, *Myo7a^Sh1/Sh1^* mice injected with either of the dual AAVs‐*Myo7a* showed significantly improved ABR thresholds compared to un‐injected *Myo7a^Sh1/Sh1^* mice (*P* < 0.0001, Tukey's post test, two‐way ANOVA, Fig. 8*B*). Over the 12—24 kHz region, there was on average about 20—30 dB improvement in ABR threshold in AAV‐*Myo7a* injected *Myo7a^Sh1/Sh1^* mice compared to *Myo7a^Sh1/Sh1^* mice (Fig. 8*B*). These results are particularly encouraging considering that both AAV vectors almost exclusively transduce IHCs (Fig. 5*A* and *B*).

## Discussion

The acquisition of hearing function requires the correct development of the stereociliary bundles on both IHCs and OHCs, which are responsible for the mechanoelectrical transduction of acoustic stimuli into an electrical signal. This is a tightly regulated developmental process requiring the interaction of many proteins, including the unconventional myosins (Barr‐Gillespie, 2015; McGrath et al., 2017; Park & Bird, 2023). The absence of functional MYO7A has been shown to cause severe morphological defects in the stereociliary bundles beginning from pre‐hearing stages, albeit with different temporal progression depending on the mutation (Self et al., 1998). Here we found that a missense mutation in the *shaker‐1* gene (*Myo7a^Sh1^*) led to hair bundle defects evident as early as P10, and by 1 month of age almost all hair cells had only one row of stereocilia. We also showed that both sensory hair cell types failed to acquire a fully mature basolateral electrophysiological profile. Exogenous delivery of *Myo7a* using dual‐AAV vectors in *Myo7a^Sh1/Sh1^* P0–P2 pups *in vivo* was able to partially restore the staircase structure of the hair bundles and the MET current in adult IHCs, which were the main target of the AAVs used (AAV8 or AAV9‐PhP.eB). IHCs from *Myo7a^Sh1/Sh1^* mice injected with AAV‐*Myo7a* had a larger expression of the basolateral K^+^ current characteristic of mature cells (*I*_K,f_) compared to *Myo7a^Sh1/Sh1^* mice. These changes translated into improved ABR thresholds of up to about 30 dB between the 6 and 30 kHz frequency range. We propose that exogenous gene augmentation using AAV vectors *in vivo* is a suitable strategy to rescue complex morphological and functional defects present in the hair cells of a mouse model of *USH1B* syndrome. Further hearing improvements would require the use of AAVs that also target OHCs such as PHP.B (György et al., 2018; Shubina‐Oleinik et al., 2021) and possibly performing *in vivo* transduction during embryonic stages (Bedrosian et al., 2006; Hu et al., 2020; Iranfar et al., 2025), which will allow expression of the exogenous *Myo7a* prior to any morphological hair bundle defects.

### MYO7A is crucial for hair bundle development and maintenance

The precise staircase‐like architecture of stereocilia within the hair bundles of both cochlear IHCs and OHCs is established primarily during embryonic and early postnatal stages through a tightly controlled process of elongation and thickening (Vélez‐Ortega & Frolenkov, 2019). This sophisticated control over the growth of stereocilia is regulated by several actin‐binding proteins and unconventional myosin motors. Since several of these crucial proteins are required during embryonic stages, their absence leads to largely undeveloped hair bundles in cochlear hair cells, resulting in hearing loss (Fang et al., 2015; Krey et al., 2020; Tadenev et al., 2019). One of the key molecules regulating the formation of the stereocilia is MYO7A, which is highly expressed along the entire length of the stereocilia and cell body of both IHCs and OHCs (Hasson et al., 1997; Underhill et al., 2025). Most of the *Myo7a* mutations in the *shaker‐1* locus, which target the motor head of the protein, lead to profoundly disorganized hair bundles (e.g. loss of their characteristic shape and staircase structure: Kros et al., 2002; Self et al., 1998). One exception is the original *shaker‐1* mutant mouse (*Myo7a^Sh1^*) in which the hair bundles are able to acquire a normal structure but start losing the shortest rows of stereocilia prior to the onset of hearing. How exactly MYO7A promotes the development and maintenance of the stereociliary bundles is still largely unclear, although several roles have been proposed over the years.

MYO7A, like other unconventional myosin motors expressed in the mammalian cochlea (e.g. MYO3A, MYO15A and possibly MYO1), uses its motor activity to deliver key proteins to the stereocilia (Miyoshi et al., 2024; Moreland & Bird, 2022). For example, MYO7A is required for transporting the barbed‐end capping protein twinfilin 2 to the stereocilia tip, which has been shown to regulate the elongation of the shorter transducing stereocilia (Peng et al., 2009; Rzadzinska, et al., 2009) that are the primary target of the *shaker‐1* mutation. The assembly of the *USH2* protein complex at stereocilia ankle links, which are filaments connecting adjacent stereocilia at their base during development (between P2 and P9: Goodyear et al., 2005), has also been shown to require MYO7A through interaction with the scaffolding protein PDZD7 (Grati et al., 2012; Michalski, et al., 2007; Morgan et al., 2016; Zou et al., 2017). Abnormal crosslinks in *Myo7a*‐deficient mice have been suggested to impact the maintenance of hair‐bundle integrity and/or reabsorption of the stereocilia that are not fully linked to the rest of the stereocilia bundle (Hasson et al., 1997). These and other activities carried out by MYO7A, including its interactions with additional stereociliary proteins (Miyoshi et al., 2024; Moreland & Bird, 2022), show that MYO7A plays a key role in the acquisition and maintenance of the structural and functional integrity of the hair bundles, a role that has recently been shown in the mature cochlea (Underhill et al., 2025).

### Hair cell maturation requires a functional MET apparatus prior to the onset of hearing

The functional maturation of cochlear hair cells is initiated by intrinsic genetic programmes that are coordinated by several transcription factors (Pyott et al., 2024). However, these genetic programmes are influenced and guided by Ca^2+^‐dependent activity that occurs in the hair cells during a critical period of cochlear development (Ceriani et al., 2019; De Faveri et al., 2025; Johnson et al., 2011; Tritsch & Bergles, 2010). This spontaneous activity has been shown to regulate the remodelling of synapses and ion‐channel expression not only in cochlear hair cells (Carlton et al., 2023; Ceriani et al., 2019; Johnson et al., 2007, 2013) but also in cells from other systems (Moody & Bosma, 2005; Zhang & Poo, 2001), probably by modulating gene expression (Dolmetsch et al, 1997).

Although Ca^2+^‐dependent signals in the developing cochlea occur in the absence of external stimuli (De Faveri et al., 2025), they are continuously modulated by several physiological mechanisms, including the activity of the MET channel (Ceriani et al., 2025). This is because from about P7 onwards, a time when the MET apparatus of hair cells is already functional (Lelli et al., 2009; Waguespack et al., 2007), the endocochlear potential starts to build up (Li et al., 2020). This generates the driving force for a sustained depolarizing MET current, which is crucial for keeping the resting membrane potential of the hair cells close to the activation threshold of Ca^2+^‐dependent action potentials (De Faveri et al., 2025; Johnson et al., 2012). Disruption of the MET current, for example in mice carrying mutations in genes targeting either hair bundle morphology (e.g. *Eps8*) or the opening or function of the MET channels (e.g. *Pcdh15*, *Ush1c*, *Tmc1*), alters the Ca^2+^ signalling dynamics in developing hair cells, which has been shown to prevent maturation of the hair cell basolateral membrane profile. This lack of maturation, which compromises the ability of hair cells to process acoustic information, includes a failure to upregulate key ion channels characteristic of mature hair cells, such as KCNQ4 and BK channels (Corns et al., 2018; Marcotti et al., 2006; Zampini et al., 2011), and the refinement of the afferent (Lee et al., 2021; Sun et al., 2018) and efferent synapses (Corns et al., 2018). Therefore, it is likely that the abnormal basolateral profile of mature hair cells from *Myo7a^Sh1^* mice is a consequence of the altered MET apparatus in developing hair cells, rather than from a direct role of MYO7A in targeting proteins to the cell membrane.

### *In vivo* delivery of AAV‐*Myo7a* partially rescues IHC function and hearing loss in *shaker‐1* mice

Recent advances in AAV‐mediated gene therapy approaches targeting hair cell genetic defects responsible for deafness have been instrumental in advancing several preclinical trials using murine models of hereditary deafness (Amariutei et al., 2023; Petit et al., 2023). Currently, the most successful studies have implicated genes involved in exocytosis (*Vglut3*: Akil et al., 2012; *Otof*: Akil et al., 2019; Al‐Moyed et al., 2019) and mechanoelectrical transduction (*Tmc1*: Askew et al., 2015; Nist‐Lund et al., 2019). However, the rescue of deafness genes crucial for the initial growth of the hair cell stereociliary bundles such as *Eps8* (Jeng et al., 2022), *Clarin‐1* (Dulon et al., 2018) and *Sans* (Emptoz et al., 2017) have shown limited success. Similarly, a recent attempt to restore hearing using gene‐based therapy in mice with a nonsense mutation in *Myo7a* (*Myo7a^4626SB/4626SB^*), which is a model for *USH1B*, did not result in detectable hearing improvements (Lau et al., 2023). In contrast, we found that injecting AAV‐*Myo7a* into the cochlea of *Myo7a^Sh1/Sh1^* mice led to partial recovery of the hair cell hair bundle structure, restoration of basolateral membrane physiology in IHCs and an improvement in auditory thresholds (up to about 30 dB). The higher degree of hearing recovery in *Myo7a^Sh1/Sh1^* compared to *Myo7a^4626SB/4626SB^* mice is likely to be due to the latter having extensive hair bundle disruption from early postnatal stages (Holme & Steel, 2002). This further supports the idea that the effectiveness of AAV gene‐based therapies for congenital deafness is dictated largely by the level of morphological damage present at the time of treatment. For genes involved in the formation of the sensory epithelium, such as most of those implicated in *USHER* syndromes, there is a narrow therapeutic window early in development, necessitating *in utero* AAV‐mediated gene delivery, as previously demonstrated for other genes (Bedrosian et al., 2006; Hu et al., 2020). An alternative possibility would be to extend the therapeutic window by delaying the progression of the morphological dysfunction.

## Additional information

## Competing interests

The authors declare no conflicts of interest.

## Author contributions

All authors helped with the collection and analysis of the data. W.M. conceived and coordinated the study. All authors approved the final version of the manuscript. All authors agree to be accountable for all aspects of the work in ensuring that questions related to the accuracy or integrity of any part of the work are appropriately investigated and resolved. All persons designated as authors qualify for authorship, and all those who qualify for authorship are listed.

## Funding

This work was supported by the French National Research Agency, funding the France 2030 program entitled RHU AUDINNOVE (ANR‐18‐RHUS‐0007 to S.S.). This work was also supported by grants from the “Fondation pour l'Audition” (FPA IDA08 to S.S.), BBSRC (BB/Z516685/1) and Wellcome Trust (224326/Z/21/Z) to W.M.; BBSRC (BB/X000567/1) to S.L.J.; Wellcome Trust (300350/Z/23/Z) to A.J.C.; BBSRC (BB/Z514743/1) to J.‐Y.J. A.O.’C. was supported by a PhD studentship from the RNID (S56) Partnership to W.M. A.E.A. was supported by a PhD studentship from the Sheffield Neuroscience Institute to W.M. A.U. was supported by a PhD studentship from the MRC DiMeN Doctoral training Partnership to W.M. For the purpose of Open Access, the author has applied a CC BY public copyright licence to any Author Accepted Manuscript version arising from this submission.

## Supporting information


Peer Review History

## Data Availability

The data that support the findings of this study are available from the corresponding authors.

## References

[tjp70135-bib-0001] Abe, T. , Kakehata, S. , Kitani, R. , Maruya, S. , Navaratnam, D. , Santos‐Sacchi, J. , & Shinkawa, H. (2007). Developmental expression of the outer hair cell motor prestin in the mouse. Journal Membrane Biology, 215(1), 49–56. 17415610 10.1007/s00232-007-9004-5 PMC4154540

[tjp70135-bib-0002] Akil, O. , Seal, R. P. , Burke, K. , Wang, C. , Alemi, A. , During, M. , Edwards, R. H. , & Lustig, L. R. (2012). Restoration of hearing in the VGLUT3 knockout mouse using virally mediated gene therapy. Neuron, 75(2), 283–293. 22841313 10.1016/j.neuron.2012.05.019 PMC3408581

[tjp70135-bib-0003] Akil, O. , Dyka, F. , Calvet, C. , Emptoz, A. , Lahlou, G. , Nouaille, S. , Boutet de Monvel, J. , Hardelin, J. P. , Hauswirth, W. W. , Avan, P. , Petit, C. , Safieddine, S. , & Lustig, L. R. (2019). Dual AAV‐mediated gene therapy restores hearing in a DFNB9 mouse model. Proceedings of the National Academy of Sciences, 116(10), 4496–4501. 10.1073/pnas.1817537116 PMC6410774 30782832

[tjp70135-bib-0004] Al‐Moyed, H. , Cepeda, A. P. , Jung, S. , Moser, T. , Kügler, S. , & Reisinger, E. (2019). A dual‐AAV approach restores fast exocytosis and partially rescues auditory function in deaf otoferlin knock‐out mice. European Molecular Biology Organization Molecular Medicine, 11, e9396. 10.15252/emmm.201809396 PMC6328916 30509897

[tjp70135-bib-0005] Amariutei, A. E. , Jeng, J. Y. , Safieddine, S. , & Marcotti, W. (2023). Recent advances and future challenges in gene therapy for hearing loss. Royal Society Open Science, 10(6), 230644. 37325593 10.1098/rsos.230644 PMC10265000

[tjp70135-bib-0006] Askew, C. , Rochat, C. , Pan, B. , Asai, Y. , Ahmed, H. , Child, E. , Schneider, B. L. , Aebischer, P. , & Holt, J. R. (2015). Tmc gene therapy restores auditory function in deaf mice. Science Translational Medicine, 7(295), a108. 10.1126/scitranslmed.aab1996 PMC7298700 26157030

[tjp70135-bib-0007] Ballesteros, A. , Yadav, M. , Cui, R. , Kurima, K. , & Kachar, B. (2022). Selective binding and transport of protocadherin 15 isoforms by stereocilia unconventional myosins in a heterologous expression system. Scientific Report, 12(1), 13764. 10.1038/s41598-022-17757-0 PMC9374675 35962067

[tjp70135-bib-0008] Barr‐Gillespie, P. G. (2015). Assembly of hair bundles, an amazing problem for cell biology. Molecular Biology of the Cell, 26(15), 2727–2732. 26229154 10.1091/mbc.E14-04-0940 PMC4571333

[tjp70135-bib-0009] Bedrosian, J. C. , Gratton, M. A. , Brigande, J. V. , Tang, W. , Landau, J. , & Bennett, J. (2006). In vivo delivery of recombinant viruses to the fetal murine cochlea: Transduction characteristics and long‐term effects on auditory function. Molecular Therapy, 14(3), 328–335. 16765094 10.1016/j.ymthe.2006.04.003 PMC4108343

[tjp70135-bib-0010] Beurg, M. , Fettiplace, R. , Nam, J. H. , & Ricci, A. J. (2009). Localization of inner hair cell mechanotransducer channels using high‐speed calcium imaging. Nature Neuroscience, 12(5), 553–558. 19330002 10.1038/nn.2295 PMC2712647

[tjp70135-bib-0011] Carlton, A. J. , Halford, J. , Underhill, A. , Jeng, J. Y. , Avenarius, M. R. , Gilbert, M. L. , Ceriani, F. , Ebisine, K. , Brown, S. D. , Bowl, M. R. , Barr‐Gillespie, P. G. , & Marcotti, W. (2021). Loss of Baiap2l2 destabilizes the transducing stereocilia of cochlear hair cells and leads to deafness. The Journal of Physiology, 599(4), 1173–1198. 33151556 10.1113/JP280670 PMC7898316

[tjp70135-bib-0012] Carlton, A. J. , Jeng, J. Y. , Grandi, F. C. , De Faveri, F. , Ceriani, F. , De Tomasi, L. , Underhill, A. , Johnson, S. L. , Legan, K. P. , Kros, C. J. , Richardson, G. P. , Mustapha, M. , & Marcotti, W. (2023). A critical period of prehearing spontaneous Ca^2+^ spiking is required for hair‐bundle maintenance in inner hair cells. European Molecular Biology Organization Journal, 42(4), e112118. 10.15252/embj.2022112118 PMC9929643 36594367

[tjp70135-bib-0013] Carlton, A. J. , Jeng, J. Y. , Grandi, F. C. , De Faveri, F. , Amariutei, A. E. , De Tomasi, L. , O'Connor, A. , Johnson, S. L. , Furness, D. N. , Brown, S. D. M. , Ceriani, F. , Bowl, M. R. , Mustapha, M. , & Marcotti, W. (2024). BAI1 localizes AMPA receptors at the cochlear afferent post‐synaptic density and is essential for hearing. Cell Report, 43(4), 114025. 10.1016/j.celrep.2024.114025 PMC7618386 38564333

[tjp70135-bib-0014] Ceriani, F. , Hendry, A. , Jeng, J. Y. , Johnson, S. L. , Stephani, F. , Olt, J. , Holley, M. C. , Mammano, F. , Engel, J. , Kros, C. J. , Simmons, D. D. , & Marcotti, W. (2019). Coordinated calcium signalling in cochlear sensory and non‐sensory cells refines afferent innervation of outer hair cells. European Molecular Biology Organization Journal, 38(9), e99839. 10.15252/embj.201899839 PMC6484507 30804003

[tjp70135-bib-0015] Ceriani, F. , Wood, K. C. , Johnson, S. L. , Kros, C. J. , & Marcotti, W. (2025). Mechanisms driving the functional maturation of the developing mammalian auditory pathway. Current Topics in Developmental Biology, 165, 185–233. 40973230 10.1016/bs.ctdb.2025.03.005 PMC7618431

[tjp70135-bib-0016] Corns, L. F. , Johnson, S. L. , Kros, C. J. , & Marcotti, W. (2014). Calcium entry into stereocilia drives adaptation of the mechanoelectrical transducer current of mammalian cochlear hair cells. Proceedings of the National Academy of Sciences, 111(41), 14918–14923. 10.1073/pnas.1409920111 PMC4205606 25228765

[tjp70135-bib-0017] Corns, L. F. , Johnson, S. L. , Roberts, T. , Ranatunga, K. M. , Hendry, A. , Ceriani, F. , Safieddine, S. , Steel, K. P. , Forge, A. , Petit, C. , Furness, D. N. , Kros, C. J. , & Marcotti, W. (2018). Mechanotransduction is required for establishing and maintaining mature inner hair cells and regulating efferent innervation. Nature Communications, 9(1), 4015. 10.1038/s41467-018-06307-w PMC6167318 30275467

[tjp70135-bib-0018] De Faveri, F. , Ceriani, F. , & Marcotti, W. (2025). In vivo spontaneous Ca^2+^ activity in the pre‐hearing mammalian cochlea. Nature Communications, 16, 29. 10.1038/s41467-024-55519-w PMC11695946 39747044

[tjp70135-bib-0019] Dolmetsch, R. E. , Lewis, R. S. , Goodnow, C. C. , & Healy, J. I. (1997). Differential activation of transcription factors induced by Ca^2+^ response amplitude and duration. Nature, 386(6627), 855–858. 9126747 10.1038/386855a0

[tjp70135-bib-0020] Dulon, D. , Papal, S. , Patni, P. , Cortese, M. , Vincent, P. F. , Tertrais, M. , Emptoz, A. , Tlili, A. , Bouleau, Y. , Michel, V. , Delmaghani, S. , Aghaie, A. , Pepermans, E. , Alegria‐Prevot, O. , Akil, O. , Lustig, L. , Avan, P. , Safieddine, S. , Petit, C. , & El‐Amraoui, A. (2018). Clarin‐1 gene transfer rescues auditory synaptopathy in model of Usher syndrome. Journal Clinical Investigation, 128(8), 3382–3401. 10.1172/JCI94351 PMC6063508 29985171

[tjp70135-bib-0021] Emptoz, A. , Michel, V. , Lelli, A. , Akil, O. , Boutet de Monvel, J. , Lahlou, G. , Meyer, A. , Dupont, T. , Nouaille, S. , Ey, E. , Franca de Barros, F. , Beraneck, M. , Dulon, D. , Hardelin, J. P. , Lustig, L. , Avan, P. , Petit, C. , & Safieddine, S. (2017). Local gene therapy durably restores vestibular function in a mouse model of Usher syndrome type 1G. Proceedings of the National Academy of Sciences, 114(36), 9695–9700. 10.1073/pnas.1708894114 PMC5594693 28835534

[tjp70135-bib-0022] Fang, Q. , Indzhykulian, A. A. , Mustapha, M. , Riordan, G. P. , Dolan, D. F. , Friedman, T. B. , Belyantseva, I. A. , Frolenkov, G. I. , Camper, S. A. , & Bird, J. E. (2015). The 133‐kDa N‐terminal domain enables myosin 15 to maintain mechanotransducing stereocilia and is essential for hearing. eLife, 4, e08627. 26302205 10.7554/eLife.08627 PMC4592939

[tjp70135-bib-0023] Fettiplace, R. , & Kim, K. X. (2014). The physiology of mechanoelectrical transduction channels in hearing. Physiological Reviews, 94(3), 951–986. 24987009 10.1152/physrev.00038.2013 PMC4101631

[tjp70135-bib-0024] Furness, D. N. , & Hackney, C. M. (1986). High‐resolution scanning‐electron microscopy of stereocilia using the osmium‐thiocarbohydrazide coating technique. Hearing Research, 21(3), 243–249. 3522517 10.1016/0378-5955(86)90222-4

[tjp70135-bib-0025] Gibson, F. , Walsh, J. , Mburu, P. , Varela, A. , Brown, K. A. , Antonio, M. , Beisel, K. W. , Steel, K. P. , & Brown, S. D. (1995). A type VII myosin encoded by the mouse deafness gene shaker‐1. Nature, 374(6517), 62–64. 7870172 10.1038/374062a0

[tjp70135-bib-0026] Goode, B. L. , Drubin, D. G. , & Barnes, G. (2000). Functional cooperation between the microtubule and actin cytoskeletons. Current Opinion Cell Biology, 12(1), 63–71. 10.1016/s0955-0674(99)00058-7 10679357

[tjp70135-bib-0027] Goodyear, R. J. , Marcotti, W. , Kros, C. J. , & Richardson, G. P. (2005). Development and properties of stereociliary link types in hair cells of the mouse cochlea. Journal Comparative Neurology, 485(1), 75–85. 10.1002/cne.20513 15776440

[tjp70135-bib-0028] Grati, M. , Shin, J. B. , Weston, M. D. , Green, J. , Bhat, M. A. , Gillespie, P. G. , & Kachar, B. (2012). Localization of PDZD7 to the stereocilia ankle‐link associates this scaffolding protein with the Usher syndrome protein network. Journal of Neuroscience, 32(41), 14288–14293. 23055499 10.1523/JNEUROSCI.3071-12.2012 PMC3518401

[tjp70135-bib-0029] György, B. , Meijer, E. J. , Ivanchenko, M. V. , Tenneson, K. , Emond, F. , Hanlon, K. S. , Indzhykulian, A. A. , Volak, A. , Karavitaki, K. D. , Tamvakologos, P. I. , Vezina, M. , Berezovskii, V. K. , Born, R. T. , O'Brien, M. , Lafond, J. F. , Arsenijevic, Y. , Kenna, M. A. , Maguire, C. A. , & Corey, D. P. (2018). Gene transfer with AAV9‐PHP.B rescues hearing in a mouse model of Usher Syndrome 3A and transduces hair cells in a non‐human primate. Molecular Therapy Methods & Clinical Development, 13, 1–13. 30581889 10.1016/j.omtm.2018.11.003 PMC6297893

[tjp70135-bib-0030] Hasson, T. , Gillespie, P. G. , Garcia, J. A. , MacDonald, R. B. , Zhao, Y. , Yee, A. G. , Mooseker, M. S. , & Corey, D. P. (1997). Unconventional myosins in inner‐ear sensory epithelia. Journal of Cell Biology, 137(6), 1287–1307. 9182663 10.1083/jcb.137.6.1287 PMC2132524

[tjp70135-bib-0030a] Hasson, T. , Heintzelman, M. B. , Santos‐Sacchi, J. , Corey, D. P. , & Mooseker, M. S. (1995). Expression in cochlea and retina of myosin VIIa, the gene product defective in Usher syndrome type 1B. Proceedings of the National Academy of Sciences of the United States of America, 92(21), 9815–9819. 7568224 10.1073/pnas.92.21.9815 PMC40893

[tjp70135-bib-0031] Holme, R. H. , & Steel, K. P. (2002). Stereocilia defects in waltzer (Cdh23), shaker1 (Myo7a) and double waltzer/shaker1 mutant mice. Hearing Research, 169(1–2), 13–23. 12121736 10.1016/s0378-5955(02)00334-9

[tjp70135-bib-0032] Hu, C. J. , Lu, Y. C. , Tsai, Y. H. , Cheng, H. Y. , Takeda, H. , Huang, C. Y. , Xiao, R. , Hsu, C. J. , Tsai, J. W. , Vandenberghe, L. H. , Wu, C. C. , & Cheng, Y. F. (2020). Efficient in utero gene transfer to the mammalian inner ears by the synthetic adeno‐associated viral vector Anc80L65. Molecular Therapy Methods & Clinical Development, 18, 493–500. 32775487 10.1016/j.omtm.2020.06.019 PMC7390729

[tjp70135-bib-0033] Ingham, N. J. , Pearson, S. , & Steel, K. P. (2011). Using the auditory brainstem response (ABR) to determine sensitivity of hearing in mutant mice. Current Protocols in Mouse Biology, 1(2), 279–287. 26069055 10.1002/9780470942390.mo110059

[tjp70135-bib-0034] Iranfar, S. , Cornille, M. , Roldan, M. S. , Plion, B. , Lecomte, M. J. , Safieddine, S. , & Lahlou, G. (2025). Cell tropism of adeno‐associated viruses within the mouse inner ear in vivo: From embryonic to adult stages. Scientific Reports, 15(1), 13479. 40251388 10.1038/s41598-025-98007-x PMC12008179

[tjp70135-bib-0035] Jeng, J. Y. , Carlton, A. J. , Goodyear, R. J. , Chinowsky, C. , Ceriani, F. , Johnson, S. L. , Sung, T. C. , Dayn, Y. , Richardson, G. P. , Bowl, M. R. , Brown, S. D. , Manor, U. , & Marcotti, W. (2022). AAV‐mediated rescue of Eps8 expression in vivo restores hair‐cell function in a mouse model of recessive deafness. Molecular Therapy Methods & Clinical Development, 26, 355–370. 36034774 10.1016/j.omtm.2022.07.012 PMC9382420

[tjp70135-bib-0036] Johnson, S. L. , Adelman, J. P. , & Marcotti, W. (2007). Genetic deletion of SK2 channels in mouse inner hair cells prevents the developmental linearization in the Ca^2+^ dependence of exocytosis. The Journal of Physiology, 583(2), 631–646. 17627990 10.1113/jphysiol.2007.136630 PMC2096744

[tjp70135-bib-0037] Johnson, S. L. , Eckrich, T. , Kuhn, S. , Zampini, V. , Franz, C. , Ranatunga, K. M. , Roberts, T. P. , Masetto, S. , Knipper, M. , Kros, C. J. , & Marcotti, W. (2011). Position‐dependent patterning of spontaneous action potentials in immature cochlear inner hair cells. Nature Neuroscience, 14(6), 711–717. 21572434 10.1038/nn.2803 PMC3103712

[tjp70135-bib-0038] Johnson, S. L. , Kennedy, H. J. , Holley, M. C. , Fettiplace, R. , & Marcotti, W. (2012). The resting transducer current drives spontaneous activity in prehearing mammalian cochlear inner hair cells. Journal of Neuroscience, 32(31), 10479–10483. 22855797 10.1523/JNEUROSCI.0803-12.2012 PMC3428842

[tjp70135-bib-0039] Johnson, S. L. , Kuhn, S. , Franz, C. , Ingham, N. , Furness, D. N. , Knipper, M. , Steel, K. P. , Adelman, J. P. , Holley, M. C. , & Marcotti, W. (2013). Presynaptic maturation in auditory hair cells requires a critical period of sensory‐independent spiking activity. Proceedings of the National Academy of Sciences, 110(21), 8720–8725. 10.1073/pnas.1219578110 PMC3666720 23650376

[tjp70135-bib-0040] Krey, J. F. , Chatterjee, P. , Dumont, R. A. , O'Sullivan, M. , Choi, D. , Bird, J. E. , & Barr‐Gillespie, P. G. (2020). Mechanotransduction‐dependent control of stereocilia dimensions and row identity in inner hair cells. Current Biology, 30(3), 442–454.e7. 31902726 10.1016/j.cub.2019.11.076 PMC7002276

[tjp70135-bib-0041] Kolla, L. , Kelly, M. C. , Mann, Z. F. , Anaya‐Rocha, A. , Ellis, K. , Lemons, A. , Palermo, A. T. , So, K. S. , Mays, J. C. , Orvis, J. , Burns, J. C. , Hertzano, R. , Driver, E. C. , & Kelley, M. W. (2020). Characterization of the development of the mouse cochlear epithelium at the single cell level. Nature Communications, 11(1), 2389. 10.1038/s41467-020-16113-y PMC7221106 32404924

[tjp70135-bib-0042] Kros, C. J. , Ruppersberg, J. P. , & Rüsch, A. (1998). Expression of a potassium current in inner hair cells during development of hearing in mice. Nature, 394(6690), 281–284. 9685158 10.1038/28401

[tjp70135-bib-0043] Kros, C. J. , Marcotti, W. , van Netten, S. M. , Self, T. J. , Libby, R. T. , Brown, S. D. , Richardson, G. P. , & Steel, K. P. (2002). Reduced climbing and increased slipping adaptation in cochlear hair cells of mice with Myo7a mutations. Nature Neuroscience, 5(1), 41–47. 11753415 10.1038/nn784

[tjp70135-bib-0044] Kubisch, C. , Schroeder, B. C. , Friedrich, T. , Lütjohann, B. , El‐Amraoui, A. , Marlin, S. , Petit, C. , & Jentsch, T. J. (1999). KCNQ4, a novel potassium channel expressed in sensory outer hair cells, is mutated in dominant deafness. Cell, 96(3), 437–446. 10025409 10.1016/s0092-8674(00)80556-5

[tjp70135-bib-0045] Lau, S. C. , Grati, M. , Isgrig, K. , Sinan, M. , Calabro, K. R. , Zhu, J. , Ishibashi, Y. , Ozgur, Z. , Wafa, T. , Belyantseva, I. A. , Fitzgerald, T. , Friedman, T. B. , Boye, S. L. , Boye, S. E. , & Chien, W. W. (2023). Dual‐AAV vector‐mediated expression of MYO7A improves vestibular function in a mouse model of Usher syndrome 1B. Molecular Therapy Methods & Clinical Development, 30, 534–545. 37693946 10.1016/j.omtm.2023.08.012 PMC10491803

[tjp70135-bib-0046] Lee, J. , Kawai, K. , Holt, J. R. , & Géléoc, G. S. (2021). Sensory transduction is required for normal development and maturation of cochlear inner hair cell synapses. eLife, 10, e69433. 34734805 10.7554/eLife.69433 PMC8598158

[tjp70135-bib-0047] Lelli, A. , Asai, Y. , Forge, A. , Holt, J. R. , & Géléoc, G. S. (2009). Tonotopic gradient in the developmental acquisition of sensory transduction in outer hair cells of the mouse cochlea. Journal of Neurophysiology, 101(6), 2961–2973. 19339464 10.1152/jn.00136.2009 PMC2694104

[tjp70135-bib-0048] Li, Y. , Liu, H. , Zhao, X. , & He, D. Z. (2020). Endolymphatic potential measured from developing and adult mouse inner ear. Frontiers in Cellular Neuroscience, 14, 584928. 33364922 10.3389/fncel.2020.584928 PMC7750192

[tjp70135-bib-0049] Libby, R. T. , & Steel, K. P. (2001). Electroretinographic anomalies in mice with mutations in Myo7a, the gene involved in human Usher syndrome type 1B. Investigative Ophthalmology & Visual Science, 42(3), 770–778. 11222540

[tjp70135-bib-0050] Lingle, C. J. , Martinez‐Espinosa, P. L. , Yang‐Hood, A. , Boero, L. E. , Payne, S. , Persic, D. , V‐Ghaffari, B. , Xiao, M. , Zhou, Y. , Xia, X. M. , Pyott, S. J. , & Rutherford, M. A. (2019). LRRC52 regulates BK channel function and localization in mouse cochlear inner hair cells. Proceedings of the National Academy of Sciences, 116(37), 18397–18403. 10.1073/pnas.1907065116 PMC6744894 31451634

[tjp70135-bib-0051] Liu, X. Z. , Walsh, J. , Mburu, P. , Kendrick‐Jones, J. , Cope, M. J. , Steel, K. P. , & Brown, S. D. (1997). Mutations in the myosin VIIA gene cause non‐syndromic recessive deafness. Nature Genetics, 16(2), 188–190. 9171832 10.1038/ng0697-188

[tjp70135-bib-0052] Lord, E. M. , & Gates, W. H. (1929). Shaker, a new mutation of the house mouse (*Mus musculus*). American Naturalist, 63(688), 435–442.

[tjp70135-bib-0052a] Marcotti, W. , Géléoc, G. S. , Lennan, G. W. , & Kros, C. J. (1999). Transient expression of an inwardly rectifying potassium conductance in developing inner and outer hair cells along the mouse cochlea. Pflugers Archiv : European Journal of Physiology, 439(1–2), 113–122. 10651007 10.1007/s004249900157

[tjp70135-bib-0053] Marcotti, W. , & Kros, C. J. (1999). Developmental expression of the potassium current IK,n contributes to maturation of mouse outer hair cells. The Journal of Physiology, 520(3), 653–660. 10545133 10.1111/j.1469-7793.1999.00653.x PMC2269630

[tjp70135-bib-0054] Marcotti, W. , Johnson, S. L. , Holley, M. C. , & Kros, C. J. (2003). Developmental changes in the expression of potassium currents of embryonic, neonatal and mature mouse inner hair cells. The Journal of Physiology, 548(2), 383–400. 12588897 10.1113/jphysiol.2002.034801 PMC2342842

[tjp70135-bib-0055] Marcotti, W. , Johnson, S. L. , & Kros, C. J. (2004). Effects of intracellular stores and extracellular Ca^2^ ^+^ on Ca^2+^‐activated K^+^ currents in mature mouse inner hair cells. The Journal of Physiology, 557(2), 613–633. 15064328 10.1113/jphysiol.2003.060137 PMC1665097

[tjp70135-bib-0056] Marcotti, W. , Erven, A. , Johnson, S. L. , Steel, K. P. , & Kros, C. J. (2006). Tmc1 is necessary for normal functional maturation and survival of inner and outer hair cells in the mouse cochlea. The Journal of Physiology, 574(3), 677–698. 16627570 10.1113/jphysiol.2005.095661 PMC1817746

[tjp70135-bib-0057] Mburu, P. , Liu, X. Z. , Walsh, J. , Saw, D., Jr , Cope, M. J. , Gibson, F. , Kendrick‐Jones, J. , Steel, K. P. , & Brown, S. D. (1997). Mutation analysis of the mouse myosin VIIA deafness gene. Gene Function, 1(3), 191–203. 10.1046/j.1365-4624.1997.00020.x 9680294

[tjp70135-bib-0058] McGrath, J. , Roy, P. , & Perrin, B. J. (2017). Stereocilia morphogenesis and maintenance through regulation of actin stability. Seminars in Cell & Developmental Biology, 65, 88–95. 27565685 10.1016/j.semcdb.2016.08.017 PMC5323431

[tjp70135-bib-0059] Michalski, N. , Michel, V. , Bahloul, A. , Lefèvre, G. , Barral, J. , Yagi, H. , Chardenoux, S. , Weil, D. , Martin, P. , Hardelin, J. P. , Sato, M. , & Petit, C. (2007). Molecular characterization of the ankle‐link complex in cochlear hair cells and its role in the hair bundle functioning. Journal of Neuroscience, 27(24), 6478–6488. 17567809 10.1523/JNEUROSCI.0342-07.2007 PMC6672440

[tjp70135-bib-0060] Miyoshi, T. , Belyantseva, I. A. , Sajeevadathan, M. , & Friedman, T. B. (2024). Pathophysiology of human hearing loss associated with variants in myosins. Frontiers in Physiology, 15, 1374901. 38562617 10.3389/fphys.2024.1374901 PMC10982375

[tjp70135-bib-0061] Moody, W. J. , & Bosma, M. M. (2005). Ion channel development, spontaneous activity, and activity‐dependent development in nerve and muscle cells. Physiological Review, 85(3), 883–941 10.1152/physrev.00017.2004 15987798

[tjp70135-bib-0062] Moreland, Z. G. , & Bird, J. E. (2022). Myosin motors in sensory hair bundle assembly. Current Opinion in Cell Biology, 79, 102132. 36257241 10.1016/j.ceb.2022.102132 PMC10230610

[tjp70135-bib-0063] Morgan, C. P. , Krey, J. F. , Grati, M. , Zhao, B. , Fallen, S. , Kannan‐Sundhari, A. , Liu, X. Z. , Choi, D. , Müller, U. , & Barr‐Gillespie, P. G. (2016). PDZD7‐MYO7A complex identified in enriched stereocilia membranes. eLife, 5, e18312. 27525485 10.7554/eLife.18312 PMC5005036

[tjp70135-bib-0064] Nist‐Lund, C. A. , Pan, B. , Patterson, A. , Asai, Y. , Chen, T. , Zhou, W. , Zhu, H. , Romero, S. , Resnik, J. , Polley, D. B. , Géléoc, G. S. , & Holt, J. R. (2019). Improved TMC1 gene therapy restores hearing and balance in mice with genetic inner ear disorders. Nature Communications, 10(1), 236. 10.1038/s41467-018-08264-w PMC6342993 30670701

[tjp70135-bib-0065] O'Connor, A. P. , Amariutei, A. E. , Zanella, A. , Hool, S. A. , Carlton, A. J. , Kong, F. , Saenz‐Roldan, M. , Jeng, J. Y. , Lecomte, M. J. , Johnson, S. L. , Safieddine, S. , & Marcotti, W. (2024). In vivo AAV9‐Myo7a gene rescue restores hearing and cholinergic efferent innervation in inner hair cells. Journal Clinical Investigation Insight, 9(23), e182138. 10.1172/jci.insight.182138 PMC11623941 39641274

[tjp70135-bib-0066] Oliver, D. , Knipper, M. , Derst, C. , & Fakler, B. (2003). Resting potential and submembrane calcium concentration of inner hair cells in the isolated mouse cochlea are set by KCNQ‐type potassium channels. Journal of Neuroscience, 23(6), 2141–2149. 12657673 10.1523/JNEUROSCI.23-06-02141.2003 PMC6742048

[tjp70135-bib-0067] Park, J. , & Bird, J. E. (2023). The actin cytoskeleton in hair bundle development and hearing loss. Hearing Research, 436, 108817. 37300948 10.1016/j.heares.2023.108817 PMC10408727

[tjp70135-bib-0068] Peng, A. W. , Belyantseva, I. A. , Hsu, P. D. , Friedman, T. B. , & Heller, S. (2009). Twinfilin 2 regulates actin filament lengths in cochlear stereocilia. Journal of Neuroscience, 29(48), 15083–15088. 19955359 10.1523/JNEUROSCI.2782-09.2009 PMC2823077

[tjp70135-bib-0069] Petit, C. , Bonnet, C. , & Safieddine, S. (2023). Deafness: From genetic architecture to gene therapy. Nature Review Genetics, 24(10), 665–686. 10.1038/s41576-023-00597-7 37173518

[tjp70135-bib-0070] Pyott, S. J. , Pavlinkova, G. , Yamoah, E. N. , & Fritzsch, B. (2024). Harmony in the molecular orchestra of hearing: Developmental mechanisms from the ear to the brain. Annual Review Neuroscience, 47(1), 1–20. 10.1146/annurev-neuro-081423-093942 PMC11787624 38360566

[tjp70135-bib-0071] Rzadzinska, A. K. , Schneider, M. E. , Davies, C. , Riordan, G. P. , & Kachar, B. (2004). An actin molecular treadmill and myosins maintain stereocilia functional architecture and self‐renewal. Journal Cell Biology, 164(6), 887–897. 10.1083/jcb.200310055 PMC2172292 15024034

[tjp70135-bib-0072] Rzadzinska, A. K. , Nevalainen, E. M. , Prosser, H. M. , Lappalainen, P. , & Steel, K. P. (2009). MyosinVIIa interacts with Twinfilin‐2 at the tips of mechanosensory stereocilia in the inner ear. PLoS ONE, 4(9), e7097. 19774077 10.1371/journal.pone.0007097 PMC2743196

[tjp70135-bib-0073] Senften, M. , Schwander, M. , Kazmierczak, P. , Lillo, C. , Shin, J. B. , Hasson, T. , Géléoc, G. S. , Gillespie, P. G. , Williams, D. , Holt, J. R. , & Müller, U. (2006). Physical and functional interaction between protocadherin 15 and myosin VIIa in mechanosensory hair cells. Journal of Neuroscience, 26(7), 2060–2071. 16481439 10.1523/JNEUROSCI.4251-05.2006 PMC2712835

[tjp70135-bib-0074] Self, T. , Mahony, M. , Fleming, J. , Walsh, J. , Brown, S. D. , & Steel, K. P. (1998). Shaker‐1 mutations reveal roles for myosin VIIA in both development and function of cochlear hair cells. Development (Cambridge, England), 125(4), 557–566. 9435277 10.1242/dev.125.4.557

[tjp70135-bib-0075] Scheffer, D. I. , Shen, J. , Corey, D. P. , & Chen, Z. Y. (2015). Gene expression by mouse inner ear hair cells during development. Journal of Neuroscience, 35(16), 6366–6380. 25904789 10.1523/JNEUROSCI.5126-14.2015 PMC4405555

[tjp70135-bib-0076] Shnerson, A. , & Pujol, R. (1982). Age‐related changes in the C57BL/6J mouse cochlea. I. Physiological findings. Brain Research, 254(1), 65–75. 10.1016/0165-3806(81)90059-6 7272773

[tjp70135-bib-0077] Shnerson, A. , Lenoir, M. , van de Water, T. R. , & Pujol, R. (1983). The pattern of sensorineural degeneration in the cochlea of the deaf shaker‐1 mouse: Ultrastructural observations. Brain Research, 9(3), 305–315. 10.1016/0165-3806(83)90028-7 6627025

[tjp70135-bib-0078] Shubina‐Oleinik, O. , Nist‐Lund, C. , French, C. , Rockowitz, S. , Shearer, A. E. , & Holt, J. R. (2021). Dual‐vector gene therapy restores cochlear amplification and auditory sensitivity in a mouse model of DFNB16 hearing loss. Science Advance, 7(51), eabi7629. 10.1126/sciadv.abi7629 PMC8673757 34910522

[tjp70135-bib-0079] Sun, S. , Babola, T. , Pregernig, G. , So, K. S. , Nguyen, M. , Su, S. M. , Palermo, A. T. , Bergles, D. E. , Burns, J. C. , & Müller, U. (2018). Hair cell mechanotransduction regulates spontaneous activity and spiral ganglion subtype specification in the auditory system. Cell, 174(5), 1247–1263.e15. 30078710 10.1016/j.cell.2018.07.008 PMC6429032

[tjp70135-bib-0080] Tadenev, A. L. D. , Akturk, A. , Devanney, N. , Mathur, P. D. , Clark, A. M. , Yang, J. , & Tarchini, B. (2019). GPSM2‐GNAI specifies the tallest stereocilia and defines hair bundle row identity. Current Biology, 29(6), 921–934.e4. 30827920 10.1016/j.cub.2019.01.051 PMC6516530

[tjp70135-bib-0081] Thurm, H. , Fakler, B. , & Oliver, D. (2005). Ca^2+^‐independent activation of BKCa channels at negative potentials in mammalian inner hair cells. The Journal of Physiology, 569(1), 137–151. 16150795 10.1113/jphysiol.2005.094680 PMC1464205

[tjp70135-bib-0082] Tritsch, N. X. , & Bergles, D. E. (2010). Developmental regulation of spontaneous activity in the mammalian cochlea. Journal of Neuroscience, 30(4), 1539–1550. 20107081 10.1523/JNEUROSCI.3875-09.2010 PMC2814371

[tjp70135-bib-0083] Underhill, A. , Webb, S. , Grandi, F. C. , Jeng, J. Y. , de Monvel, J. B. , Plion, B. , Carlton, A. J. , Amariutei, A. E. , Voulgari, N. , De Faveri, F. , Ceriani, F. , Mustapha, M. , Johnson, S. L. , Safieddine, S. , Kros, C. J. , & Marcotti, W. (2025). MYO7A is required for the functional integrity of the mechanoelectrical transduction complex in hair cells of the adult cochlea. Proceedings of the National Academy of Sciences, 122(1), e2414707122. 10.1073/pnas.2414707122 PMC11725811 39746042

[tjp70135-bib-0084] Vélez‐Ortega, A. C. , & Frolenkov, G. I. (2019). Building and repairing the stereocilia cytoskeleton in mammalian auditory hair cells. Hearing Research, 376, 47–57. 30638948 10.1016/j.heares.2018.12.012 PMC6456422

[tjp70135-bib-0085] Waguespack, J. , Salles, F. T. , Kachar, B. , & Ricci, A. J. (2007). Stepwise morphological and functional maturation of mechanotransduction in rat outer hair cells. Journal of Neuroscience, 27(50), 13890–13902. 18077701 10.1523/JNEUROSCI.2159-07.2007 PMC6673611

[tjp70135-bib-0086] Weil, D. , Blanchard, S. , Kaplan, J. , Guilford, P. , Gibson, F. , Walsh, J. , Mburu, P. , Varela, A. , Levilliers, J. , Weston, M. D. , Kelley, P. M. , Klmberling, W. J. , Wagenaar, M. , Levi‐Acobas, F. , Larget‐Piet, D. , Munnich, A. , Steel, K. P. , Brown, S. D. M. , & Petit, C. (1995). Defective myosin VIIA gene responsible for Usher syndrome type 1B. Nature, 374(6517), 60–61. 7870171 10.1038/374060a0

[tjp70135-bib-0087] Weil, D. , Küssel, P. , Blanchard, S. , Lévy, G. , Levi‐Acobas, F. , Drira, M. , Ayadi, H. , & Petit, C. (1997). The autosomal recessive isolated deafness, DFNB2, and the Usher 1B syndrome are allelic defects of the myosin‐VIIA gene. Nature Genetics, 16(2), 191–193. 9171833 10.1038/ng0697-191

[tjp70135-bib-0088] Zampini, V. , Rüttiger, L. , Johnson, S. L. , Franz, C. , Furness, D. N. , Waldhaus, J. , Xiong, H. , Hackney, C. M. , Holley, M. C. , Offenhauser, N. , Di Fiore, P. P. , Knipper, M. , Masetto, S. , & Marcotti, W. (2011). Eps8 regulates hair bundle length and functional maturation of mammalian auditory hair cells. PLoS Biology, 9(4), e1001048. 21526224 10.1371/journal.pbio.1001048 PMC3079587

[tjp70135-bib-0089] Zhang, L. I. , & Poo, M. M. (2001). Electrical activity and development of neural circuits. Nature Neuroscience, 4(S11), 1207–1214. 10.1038/nn753 11687831

[tjp70135-bib-0090] Zhao, X. , Liu, H. , Liu, H. , Cai, R. , & Wu, H. (2022). Gene therapy restores auditory functions in an adult Vglut3 knockout mouse model. Human Gene Therapy, 33(13–14), 729–739. 35726398 10.1089/hum.2022.062

[tjp70135-bib-0091] Zou, J. , Chen, Q. , Almishaal, A. , Mathur, P. D. , Zheng, T. , Tian, C. , Zheng, Q. Y. , & Yang, J. (2017). The roles of USH1 proteins and PDZ domain‐containing USH proteins in USH2 complex integrity in cochlear hair cells. Human Molecular Genetics, 26, 624–636. 28031293 10.1093/hmg/ddw421 PMC6075602

